# Unravelling the molecular mechanisms of vegetative-to-reproductive transition in *Cynara cardunculus* by RNA-Seq analysis

**DOI:** 10.1007/s11103-025-01679-2

**Published:** 2026-01-31

**Authors:** A. Paulino, I. Fernandes, R. C. Pires, A. Usié, A. Faustino, J. Santos, T. Brás, D. Rosa, O. S. Paulo, M. F. Duarte, L. Marum

**Affiliations:** 1 Alentejo Biotechnology Center for Agriculture and Agro-Food (CEBAL), Polytechnic University of Beja (IPBeja), Beja, Portugal; 2 cE3c - Centre for Ecology, Evolution and Environmental Changes and Change - Global Change and Sustainability Institute, Biology Department, Faculty of Sciences, University of Lisbon, P-1749-016 Lisboa, Portugal; 3 MED – Mediterranean Institute for Agriculture, Environment and Development and CHANGE – Global Change and Sustainability Institute,, CEBAL, Beja, Portugal; 4 MED – Mediterranean Institute for Agriculture, Environment and Development, Institute for Research and Advanced Training (IIFA), University of Évora, Ap. 94, Évora, Portugal; 5 Allelopathy Group, Department of Organic Chemistry, INBIO Institute of Biomolecules, Campus de Excelencia Internacional Agroalimentario (ceiA3), University of Cádiz, Puerto Real, Cádiz Spain

**Keywords:** Cardoon, Inflorescence development, Defense response, Cynaropicrin, Transcriptome

## Abstract

*Cynara cardunculus* inflorescence plays a significant role in cheese manufacturing and human consumption due to the milk-clotting capacity and the production of edible immature capitula. Consequently, it is crucial to understand the complex cellular and molecular processes involved in the development of cardoon flowers. The current study used western wild cardoon in two developmental stages, vegetative and reproductive, to understand the molecular mechanisms mediating cardoon`s vegetative-to-reproductive transition. Transcriptome profiling identified 552 differentially expressed genes (DEGs) between vegetative and reproductive stages, with 321 upregulated in stage 4 (vegetative state) and 231 in stages 5/6 (reproductive state). Gene Ontology (GO) analysis revealed DEGs involved in biological processes such as oxidation-reduction, lipid metabolism, and defense responses. Metabolic pathways, including phenylpropanoid biosynthesis and anther/pollen development, were significantly enriched. Genes associated with lignin formation, flavonoid synthesis, and acetyltransferase activity were upregulated in the vegetative stage. At the same time, those related to flower development and sesquiterpene lactone biosynthesis were prominent in the reproductive stage. Transcription factors, including MADS-box and ethylene-responsive families, were critical in regulating developmental transitions. This study provides comprehensive insights into the morphological, chemical, and molecular dynamics of *C. cardunculus* across its phenological stages, underscoring the plant’s adaptability and potential for pharmaceutical and industrial applications. The findings highlight the importance of seasonal and developmental timing in optimizing the production of valuable secondary metabolites, such as cynaropicrin.

**Supplementary Information:**

The online version contains supplementary material available at 10.1007/s11103-025-01679-2.

## Key message

Understanding molecular changes in *Cynara cardunculus* during flowering reveals key genes and pathways, aiding flower development and metabolite production, with applications in cheese production, pharmaceuticals, and industry.

**Supplementary Information:**

The online version contains supplementary material available at 10.1007/s11103-025-01679-2.

## Introduction

*Cynara cardunculus* L., commonly known as cardoon, is a perennial herbaceous plant native to the Mediterranean Basin and comprises a complex of wild and cultivated forms that are ecologically and economically significant. Belongs to the Compositae (Asteraceae), the largest and most successful dicot botanical family (Portis et al. 2019) and is valued for its applications in the pharmaceutical, food, and bioenergy industries (Raccuia and Melilli 2007). Taxonomically, this species includes the wild type (*C. cardunculus* var. *sylvestris*) with distinctive morphological types (eastern and western wild cardoons), and two cultivated varieties, *C. cardunculus* var. *altilis* (cultivated cardoon) and *C. cardunculus* var. *scolymus* (globe artichoke) (Wiklund 1992; Rottenberg and Zohary 1996; Sonnante et al. 2007; Gatto et al. 2013).

The eastern wild cardoon (*C. cardunculus* subsp. *cardunculus*) is predominantly distributed in the eastern Mediterranean region, including Greece, Cyprus, southern Italy, Sicily, and Sardinia. This subspecies exhibits reduced plants and floral heads, as well as elongated spiny bracts, and is widely regarded as the ancestral progenitor of cultivated artichoke (*C. cardunculus* var. *scolymus*) (Rottenberg and Zohary 1996). In contrast, western wild cardoon (*C. cardunculus* subsp. Flavescens Wiklund), distributed throughout the western Mediterranean, including Portugal, Spain, France, and the Canary Islands consists of more robust individuals with yellow-margined bracts and fewer, shorter spines (Gatto et al. 2013; Castro et al. 2021). It is hypothesized to be genetically closer to cultivated cardoon (*C. cardunculus* var. *altilis*), possibly representing an intermediate form between wild and domesticated types (Wiklund 1992; Sonnante et al. 2008; Gatto et al. 2013).

Cultivated varieties of *C. cardunculus* are of high agricultural value. *C. cardunculus* var. *altilis* is primarily grown for its edible stalks in the Iberian Peninsula, southern France, and Italy, and is also used together with wild types in traditional cheese production as a source of plant-based milk coagulants (Sonnante et al. 2007). *C. cardunculus* var. *scolymus* (globe artichoke), characterized by large, tender floral heads and reduced spininess, is widely cultivated throughout Mediterranean and temperate regions and is a significant horticultural crop (Portis’ et al. 2005; Lattanzio et al. 2009; Rau et al. 2016).

*C. cardunculus* exhibits a complex annual growth cycle, developing rosette-form leaves in fall and winter, and transitioning to stalk emergence and flowering in late spring. Harvesting occurs in early summer, with seeds maturing by late summer (Gominho et al. 2011). Archontoulis et al. (2010) were the first to develop a simple coding system based on the Biologische Bundesanstalt, Bundessortenamt, CHemische Industrie (BBCH) scale for *C. cardunculus* phenological growth stages, identifying several major stages, including the differentiation of the vegetative phase from stages 0 to 4, and the reproductive phase from stages 5 to 9 (Archontoulis et al. 2010). Like other flowering plants, its inflorescence development is complex and highly regulated, involving intricate molecular and cellular events and significant genetic and environmental interactions (Harder and Prusinkiewicz 2013). Furthermore, the transition from vegetative to reproductive stages involves complex genetic networks that coordinate flower development, defense responses, and secondary metabolite synthesis (Krizek and Fletcher 2005). Secondary metabolites play a crucial role in protecting floral development by mediating responses to biotic and abiotic stressors and influencing flower morphology and differentiation (Divekar et al. 2022). Our research team has described the production of secondary metabolites, such as cynaropicrin, for the first time (Ramos et al. 2013, 2019). Cynaropicrin has an enormous biological potential (Velez et al. 2012; Brás et al. 2015; Ramos et al. 2017) with bioactive properties (Eljounaidi et al. 2015), which vary significantly across developmental stages and genotypes, highlighting the importance of seasonal and phenological monitoring (Petropoulos et al. 2019). Understanding the molecular events underlying the reproductive transition in cardoon is essential for its effective breeding and conservation. As far as we know, to date, only one transcriptomic study has been carried out in cultivated cardoon, revealing the first insights of gene expression dynamics associated with the biosynthesis of valuable secondary metabolites such as phenylpropanoids, flavonoids, and sesquiterpene lactones during inflorescence development (Puglia et al. 2020).

Therefore, the present study aimed to assess for the first time the differences in gene expression patterns between the vegetative and reproductive phases using an RNA-Seq approach, providing new insights into the regulatory networks of flowering and secondary metabolites biosynthesis of wild cardoon, namely in western type.

## Materials and methods

### Plant material and morphological evaluation

To evaluate the developmental stage (transition from vegetative to reproductive growth), seven genotypes of western wild cardoons (*C. cardunculus* L. subsp. *flavescens* Wilk.) located in experimental fields in Beja, Portugal (37.70622, − 8.08808), were monitored and morphologically evaluated using the BBCH scale (Archontoulis et al. 2010), from the beginning of spring (March) until the end of May 2020. Based on this morphological evaluation, leaf samples of the two developmental stages (stage 4, corresponding to the development of harvestable vegetative plant parts, and stages 5/6, corresponding to the inflorescence emergence and development (stage 5) and flowering and capitulum formation (stage 6) were collected. Plant height, foliar area, and number of inflorescences metrics were used to evaluate plant growth for each seasonal period.

The plant material was collected in 2020 during two seasonal periods (March, stage 4; and May, stage 5/6) for chemical and molecular analyses. The plant material was air-dried at room temperature until dry, and then ground with a home grinder (Moulinex) for chemical procedures. At the same time, samples for RNA extraction were flash-frozen in liquid nitrogen on-site and kept at − 80 °C until use.

### Cynaropicrin quantification

To evaluate the biochemical changes associated with flowering and capitulum formation in wild cardoon, the sesquiterpene lactone cynaropicrin was examined at stages 4 and 5/6 of development. Cynaropicrin extraction was performed according to the previously described (Brás et al. 2020). The cynaropicrin concentration of *C. cardunculus* leaf extracts was determined using High-Performance Liquid Chromatography (HPLC, Dionex Ultimate 3000, Thermo Scientific, Waltham, Massachusetts, U.S.) with a standard curve constructed from known concentrations of the compound (0.1–0.5 mg/mL). The curve showed excellent linearity (R^2^ = 0.997) (Fig. S1). Ethanol absolute anhydrous for analysis (Carlos Herba Reagents, France) was used for extraction experiments, and Cynaropicrin concentration was analyzed with Milli Q water and acetonitrile (> = 99,9%), HPLC gradient grade (Fisher Chemical). Cynaropicrin (> = 97,5% purity) was obtained from Extrasynthese, Genay, France. The results were given in mg/g of dry weight (DW).

### RNA extraction and sequencing

Total RNA was extracted from *C. cardunculus* fresh leaves sampled using the RNAqueous-4PCR RNA Isolation kit (InvitrogenTM), according to the manufacturer's protocol with minor modifications. The concentration and integrity of RNA were evaluated by UV–Vis spectrophotometer (NanoVueplus, GE Healthcare Life Sciences, USA), agarose gel electrophoresis (1%), and Agilent BioAnalyzer system (BioRad, USA). High-quality RNA was processed for stranded paired-end (PE) cDNA library construction (DNBSEQ Transcriptome), which was then subjected to sequencing through the DNBseq platform to produce PE reads of 150 bps in length at BGI (Shenzhen, China).

### RNA sequencing data analysis

Raw reads were subjected to a quality evaluation using FastQC v.0.11.9 (Andrews 2010) and MultiQC v.1.12 (Ewels et al. 2016) to better define the quality thresholds to apply. Pre-processing was performed with Trimmomatic v.0.39 (Bolger et al. 2014) to remove adaptor/primer contaminations and low-quality reads with the following parameters: *ILLUMINACLIP:TruSeq3-PE-3.fa:2:30:10:2:keepBothReads MINLEN:36*. Before running FastQ Screen v.0.14.0 (Wingett and Andrews 2018) to check for contaminants against the genome of the most common model organisms, the reference genome of *C. cardunculus* v.2 was downloaded from the Global Artichoke Genome Database (http://www.artichokegenome.unito.it, accessed on 9th March 2022) (Acquadro et al. 2020) and included in the set of genomes used for comparison in FastQ Screen. The reads surviving the quality procedures were mapped against the cardoon genome using STAR v.2.6.1 with default parameters (Dobin et al. 2013). Only the unique mapped reads were considered for gene quantification with Htseq-count v.1.99.2 (Anders et al. 2015). Statistical analysis of differential gene expression was performed with DESeq2 v.1.28.1 (Love et al. 2014), under R v.4.0.2 (R Core Team 2020) to identify significant differences in the relative abundance of expressed genes between different development stages. Benjamini and Hochberg’s approach was used for controlling the false discovery rate, FDR (Benjamini and Hochberg 2000). Differentially expressed genes (DEGs) were defined as those with an absolute log2 fold-change (logFC) ≥ 2 and an FDR < 0.05. The associations between DEGs and GO terms were visualized with Cytoscape v.3.9.1 (Shannon et al. 2003). Additionally, DEGs were annotated with the Combined Pathway function of OmicsBox v.3.029 (BioBam) against the Reactome and KEGG databases ( 2019).

### Phylogenetic analysis

Protein sequences of 21 biological relevant DEGs associated with the vegetative-to-reproductive transition were retrieved from the corresponding genome annotation (|log2FC|≥ 2). The 21 genes analyzed in this study were selected as representative candidates based on extreme differential expression across functional categories, encompassing processes such as secondary metabolism, transcriptional regulation, signaling, defense, and development. This strategy allowed investigation of both their evolutionary relationships and regulatory control. The sequences were aligned using MAFFT v7 (Katoh & Standley 2013), and the alignment was manually inspected to ensure the correct positioning of conserved regions. Phylogenetic analysis was performed using the Maximum Likelihood (ML) method implemented in IQ-TREE v2 (Nguyen et al. 2015). The resulting tree was visualized and edited using FigTree v1.4.4 (Rambaut 2018).

### Protein motif identification

Conserved motifs in the 21 protein sequences were identified using the MEME Suite v5.5.0 (Bailey et al. 2009). Analyses were performed in protein mode with the zero or one occurrence per sequence (ZOOPS) model. The maximum number of motifs was set to 10, with motif widths ranging from 6 to 50 amino acids. All other parameters were kept at default values. The resulting motifs were visualized as sequence logos.

### Promoter sequence and cis-regulatory element analysis

De novo motif discovery in the promoter sequences was also performed using MEME v5.5.0, in DNA mode. The ZOOPS model was applied with a maximum of 10 motifs, and motif widths ranging from 6 to 15 bp. These motifs were used to identify conserved cis-regulatory patterns among the 21 promoters. The 1500 bp promoter sequences were further analyzed using the PlantCARE database (Lescot et al. 2002) to identify putative cis-acting regulatory elements. All detected elements were classified into functional categories, including core promoter elements, hormone-responsive elements, stress-responsive elements and light-responsive elements. The total number and distribution of regulatory elements were recorded for each gene.

### Correlation analysis

To assess the relationship between metabolite accumulation and transcriptional changes, we performed a metabolite–transcriptome correlation analysis using matched cynaropicrin quantification and RNA-seq expression data from the same plants. Spearman’s rank correlations (ρ) were computed between cynaropicrin levels and the expression of all detected genes. *P*-values were adjusted using the Benjamini–Hochberg false discovery rate (FDR), and genes with |ρ|≥ 0.70 and FDR < 0.05 were considered significantly associated. Significant genes were visualised using heatmaps (pheatmap package in R).

### Statistical analysis

For morphological and chemical analysis of each plant, three samples (n = 3) were analyzed, and all the assays were carried out in triplicate. Data were analyzed with one-way analysis of variance (ANOVA), while mean comparison was performed with a t-test (*p* ≤ 0.05).

## Results

### Morphological evaluation

**Fig. 1:**
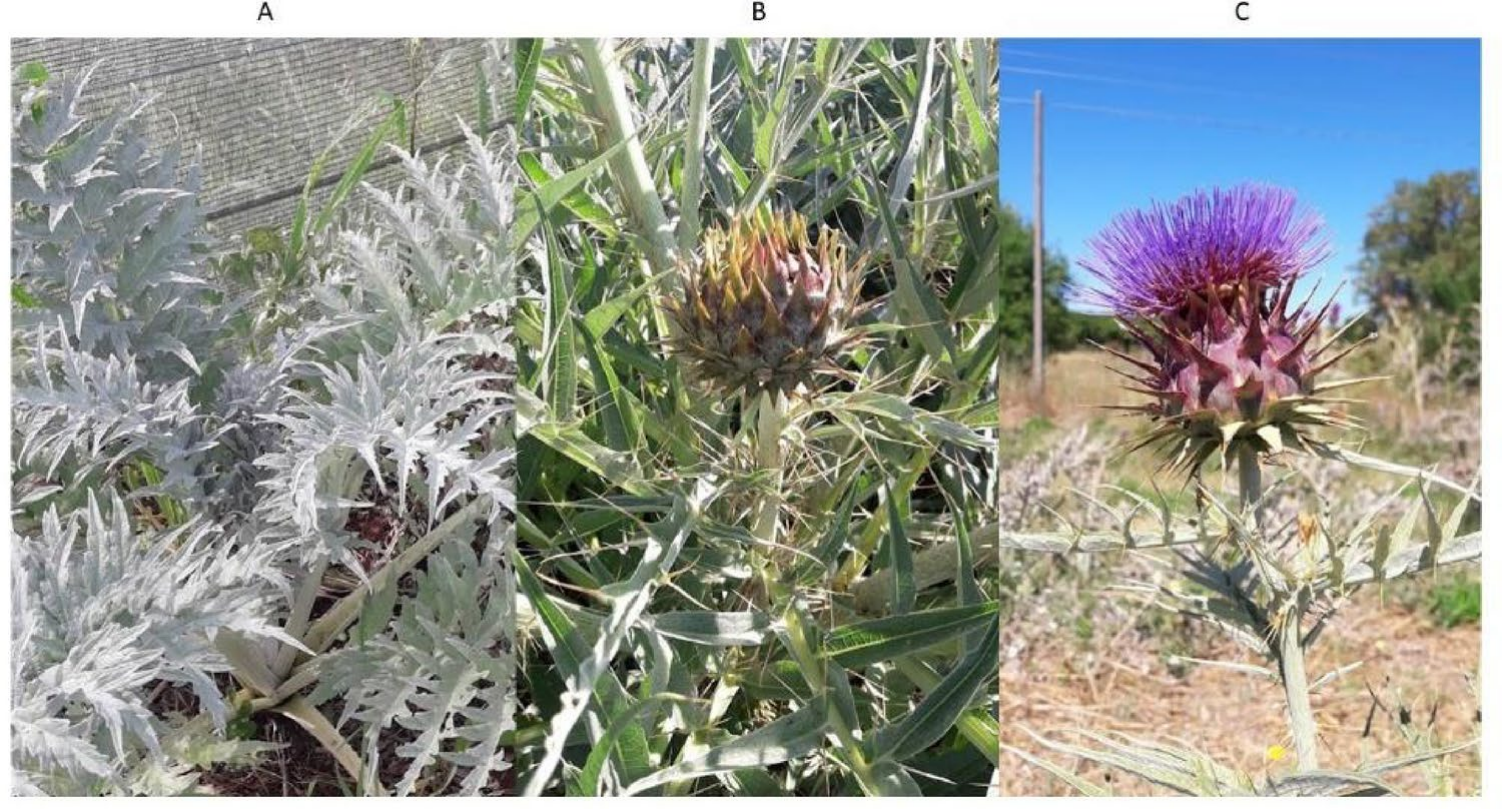
Phenological growth stages of *Cynara cardunculus* based on the BBCH scale. **A** represents stage 4, while **B** and **C** represents stages 5/6

*Cynara cardunculus* is a common perennial plant mainly distributed in the Mediterranean. The morphological evaluation was performed during pring where the vegetative to reproductive transition stages occur, according to Archontoulis et al (2010). Based on the results obtained, two sampling points were defined: (1) in March, when the adult leaves were expanded entirely and developed, and no inflorescence was emerging, being in accordance with stage 4 of BBCH scale (Fig. 1a); and (2) in May, when the emergence of inflorescences was observed, stage 5/6 of BBCH scale (Fig. 1b, c).

**Fig. 2:**
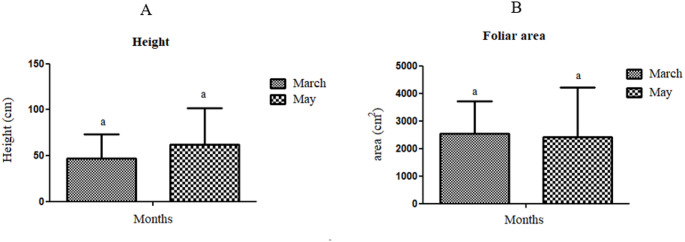
Morphological evaluation. **A** represents height (cm) while **B** represents foliar area (cm2). Each bar represents the mean ± SD (t-test, *p* > 0.05) (n = 7)

At stage 4, a maximum and a minimum height of 102 cm and 22 cm, respectively, were obtained, while the foliar area ranged from a maximum of 5062 ± 614 cm^2^ to a minimum of 1684 ± 371 cm^2^ (Table S1). At stage 5/6, it was possible to observe the emergence of inflorescences in some of the genotypes sampled. The number of inflorescences (flower buds with main and secondary capitulum) varied from 0 to 12, including immature green buds and capitulum in full flowering. The range of inflorescence types/stages that emerged may indicate some lack of synchronization in flowering time. A higher plant height mean (62 ± 40 cm) was obtained in stages 5/6 when compared with stage 4 (47 ± 26 cm), while a slight decrease in the foliar area was observed from (2540 ± 1166 cm^2^) to (2414 ± 1794 cm^2^), in stage 4 to stage5/6, respectively, despite no significant difference being detected (*p* > 0,05) (Fig. 2a, b).

### Cynaropicrin quantification

*C. cardunculus* presents several important chemical compounds, including cynaropicrin, a bitter alkaloid found in various species of the *Cynara* genus, and the most representative compound in leaves (Ramos et al. 2013) with applications in both the pharmaceutical and food industries.

The results obtained from the different plants in the two collection months of 2020 show significant variability in cynaropicrin content, ranging from 15.8 ± 1.8 to 63.1 ± 1.0, depending on the genotype and collection period (Table 1). A representative chromatogram of a cardoon sample is shown in Figure S2, where the cynaropicrin peak is clearly identified (retention time ~ 11 min).

**Table 1:** Summary of *Cynara cardunculus* samples used in transcriptome study by two developmental stages (May (stage 5/6) versus March (stage 4))

Collection	Development stage	Sample ID	Cyn (mg/g DW)
March	Stage 4	1	33,2 ± 4,1
March	Stage 4	3	15,8 ± 1,8
March	Stage 4	5	20,3 ± 1,3
March	Stage 4	7	18,8 ± 0,7
March	Stage 4	9	20,2 ± 2,0
March	Stage 4	11	23,2 ± 1,2
March	Stage 4	13	33,5 ± 0,8
May	Stage 5/6	2	37,7 ± 2,6
May	Stage 5/6	4	56,1 ± 4,2
May	Stage 5/6	6	72,5 ± 3,6
May	Stage 5/6	8	63,1 ± 1,0
May	Stage 5/6	10	60,5 ± 4,2
May	Stage 5/6	12	24,9 ± 0,5
May	Stage 5/6	14	61,8 ± 6,8

**Fig. 3:**
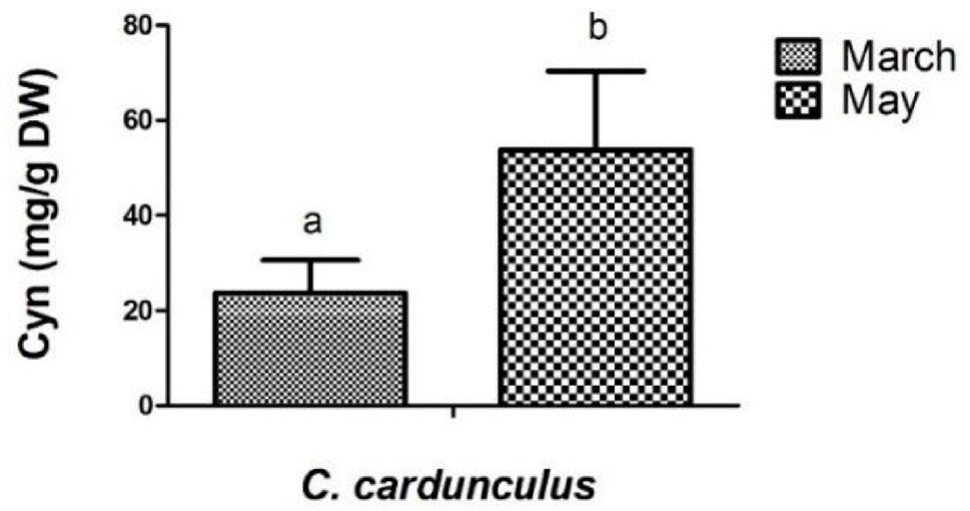
Results from cynaropicrin (Cyn) content (mg/g DW) of different Cynara cardunculus populations compared March (reproductive stage—stage 4) to May (vegetative stage—stage 5/6) 2020. Each bar represents the mean ± SD, and different letters indicate significant differences (t-test, *p* ≤ 0.05)

The cynaropicrin concentration mean in *C. cardunculus* plants was significantly higher in stage 5/6 compared to stage 4 (*p* ≤ 0,05) (Fig. 3).

### Transcriptome profiling

The sequencing run yielded approximately 47 million raw reads per sample. After applying the pre-processing procedures, 99.25% of reads were kept across the dataset, and no contamination from other organisms was detected (see Table 2 and Fig. S3). Mapping results for high-quality reads revealed that 95% of them mapped against the reference genome, with 91% being unique mapped reads (Table 2).

**Table 2:** Summary statistics of sequencing data and mapping of reads from Cc samples, identified according to each sample ID

Sample ID	Accession number (NCBI)	Raw reads (BGI)	Clean reads (%)	% Uniquely mapped reads	% multi-mapped reads	% Unmapped reads
1	SAMN35325406	46 993 406	46 626 736 (99.22)	91.62	4.66	3.72
2	SAMN35325407	48 002 372	47 616 340 (99.20)	92.06	3.34	4.60
3	SAMN35325401	46 993 378	46 636 194 (99.24)	90.70	3.03	6.27
4	SAMN35325402	41 594 992	41 221 212 (99.10)	91.86	4.53	3.61
5	SAMN35325393	47 103 680	46 737 516 (99.22)	91.61	4.79	3.60
6	SAMN35325394	47 500 532	47 154 052 (99.27)	88.39	4.25	7.36
7	SAMN35325385	46 865 244	46 500 376 (99.22)	90.99	4.53	4.47
8	SAMN35325386	47 757 296	47 354 516 (99.16)	90.98	4.49	4.53
9	SAMN35325379	47 036 012	46 789 390 (99.48)	92.12	3.88	3.99
10	SAMN35325397	46 988 390	46 617 012 (99.21)	92.28	3.72	3.99
11	SAMN35325391	47 758 670	47 399 190 (99.25)	90.58	5.03	4.38
12	SAMN35325383	47 779 774	47 400 222 (99.21)	88.42	2.72	8.86
13	SAMN35325378	47 308 424	47 077 464 (99.51)	92.48	4.57	2.95
14	SAMN35325381	47 607 874	47 249 124 (99.25)	90.06	4.11	5.83

The number of genes expressed per sample was determined using quantification data from Htseq-count, considering only genes with a minimum abundance of 5 for further analyses. Hence, the total number of genes expressed in stage 4 was 22,446, whereas in stage 5/6 it was 22,687 (Table S2).

**Fig. 4:**
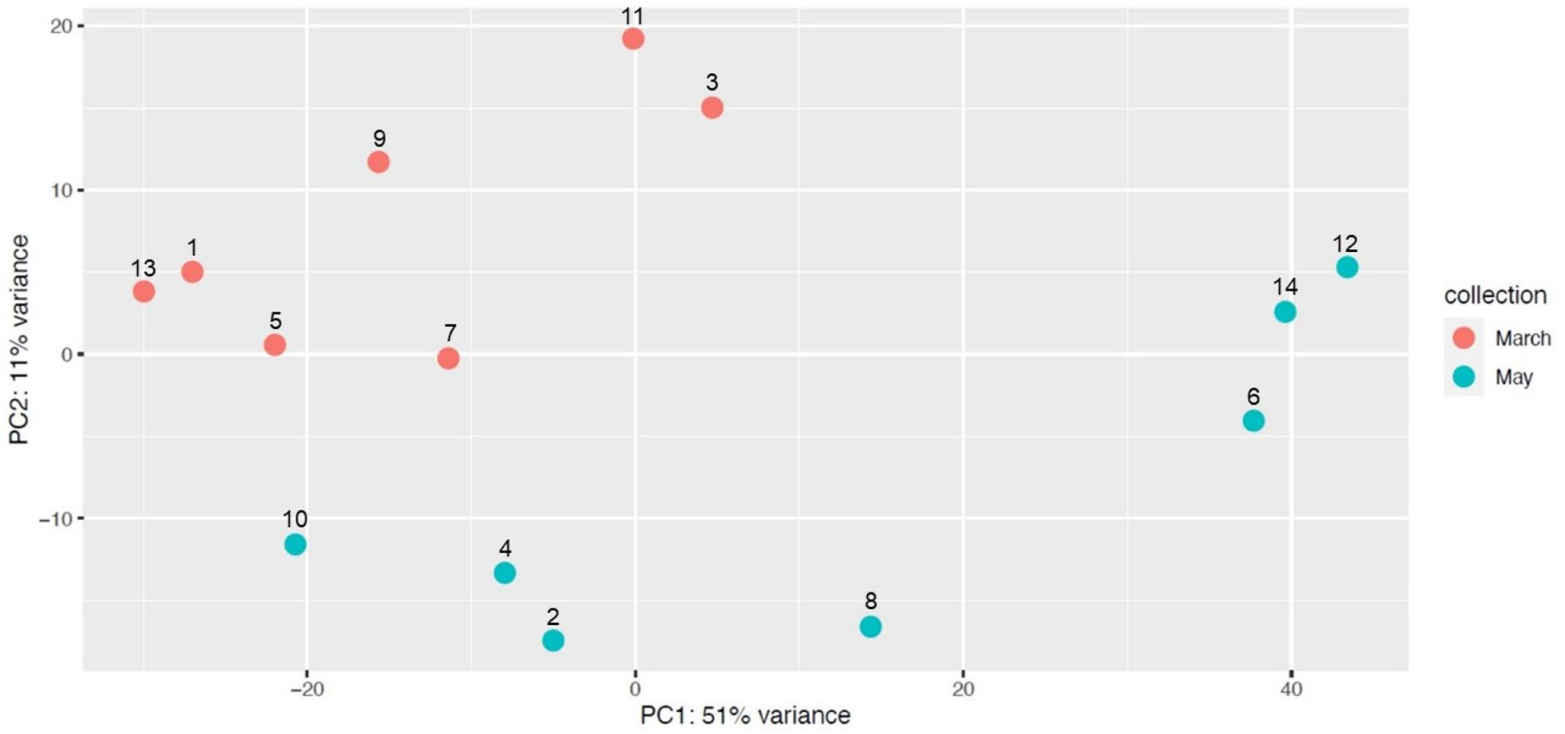
Principal component analysis (PCA) of normalized gene expression data from *Cynara cardunculus*. Two developmental stages were evaluated (May (stage 5/6) versus March (stage 4)). Colors represent different months’ collections. The percentage of variance is indicated on each axis

The PCA separates the samples from stages 4 and stage 5/6. (Fig. 4). However, the plants in stage 5/6 show a greater dispersion when compared with plants in stage 4, which could be related with in stage 4, the plants are in the vegetative development phase, whereas in stages 5/6, they transition into reproductive stages, with inflorescence emergence and development occurring in stage 5, followed by flowering and capitulum formation in stage 6.

### Identification and classification of DEGs

The gene expression profiles of stage 4 and stage 5/6 were compared to determine their expression profile differences. As a result of the analyses 552 differentially expressed genes (DEGs) were identified, from which 321 were more expressed in stage 4 and 231 in stage 5/6. The 88.0% of DEGs had a functional annotation, whereas the remaining were proteins with unknown functions. Regarding Gene Ontology (GO) terms, 383 (64.4%) DEGs had at least one GO term associated. The total number of GO represented in the DEGs set was 220 (Biological Processes: 80; Molecular Function: 119; Cellular Component: 21). The GOs associated with a higher number of differentially expressed genes are represented in Table 3.

**Table 3:** The 10% of gene ontology terms per category with a higher number of DEGs associated

Category	GO ID	GO name	# DEGs
BP	GO:0055114	Obsolete oxidation–reduction process	88
	GO:0006355	Regulation of DNA-templated transcription	30
	GO:0006629	Lipid metabolic process	21
	GO:0005975	Carbohydrate metabolic process	15
	GO:0006952	Defense response	13
	GO:0006468	Protein phosphorylation	12
	GO:0055085	Transmembrane transport	10
	GO:0006508	Proteolysis	10
MF	GO:0016491	Oxidoreductase activity	39
	GO:0020037	Heme binding	34
	GO:0005515	Protein binding	31
	GO:0005506	Iron ion binding	30
	GO:0016705	Oxidoreductase activity, acting on paired donors, with incorporation or reduction of molecular oxygen	28
	GO:0003677	DNA binding	28
	GO:0005524	ATP binding	22
	GO:0003700	DNA-binding transcription factor activity	21
	GO:0003824	Catalytic activity	19
	GO:0016788	Hydrolase activity, acting on ester bonds	16
	GO:0046983	Protein dimerization activity	14
	GO:0004672	Protein kinase activity	12
CC	GO:0016020	Membrane	16
	GO:0005634	Nucleus	9
	GO:0042025	Host cell nucleus	4

Biological process (BP), Molecular Function (MF), and cellular component (CC). #DEGs indicate the number of DEGs annotated with each GO term

The DEGs identified were mainly involved in biological processes such as obsolete oxidation–reduction process, regulation of DNA-templated transcription, lipid metabolic process, carbohydrate metabolic process, defense response, protein phosphorylation, transmembrane transport, and proteolysis. The genes involved in the molecular function were shown to be differentially regulated by oxidoreductase activity, and binding activities including the DNA-binding transcription factor (TF) activity, hydrolase activity, protein dimerization activity, and protein kinase activity.

**Fig. 5:**
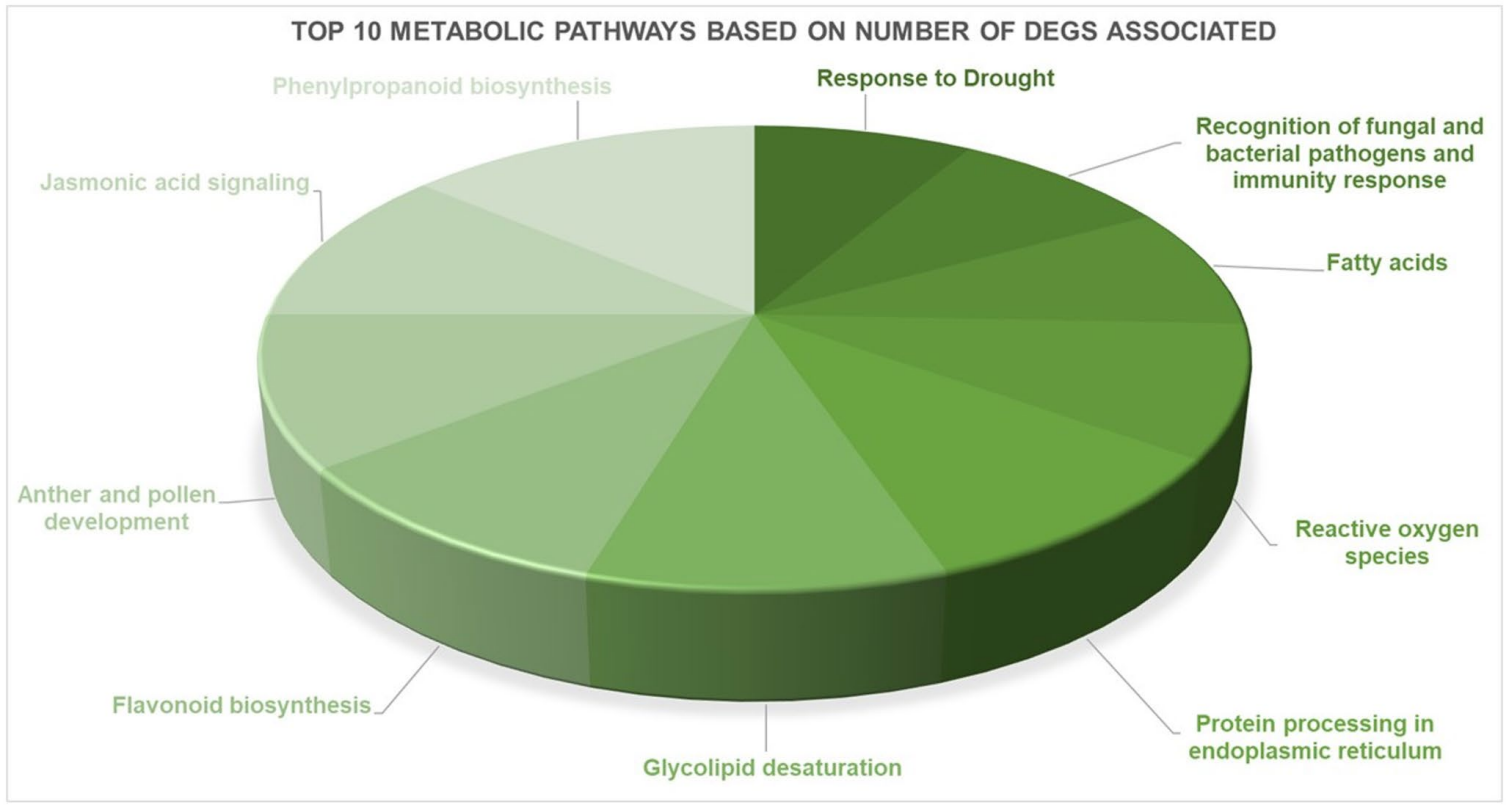
The top 10 metabolic pathways with a higher number of DEGs associated with reactome and KEGG pathways

The annotation against Reactome and KEGG pathways resulted in 378 (68.5%) DEGs associated with at least one of those metabolic pathways. The large number of DEGs related to the synthesis of plant secondary metabolites attests to their importance. The metabolic pathways with more DEGs associated are represented in Fig. 5, in which phenylpropanoid biosynthesis and anther/pollen development present some of the top metabolic pathways.

### Genes highly up-regulated in the vegetative stage (stage 4)

Due to the high number of genes identified, the |log2FC|≥ 2 was raised to enhance DEGs with the highest differences between the two developmental stages.

**Fig. 6:**
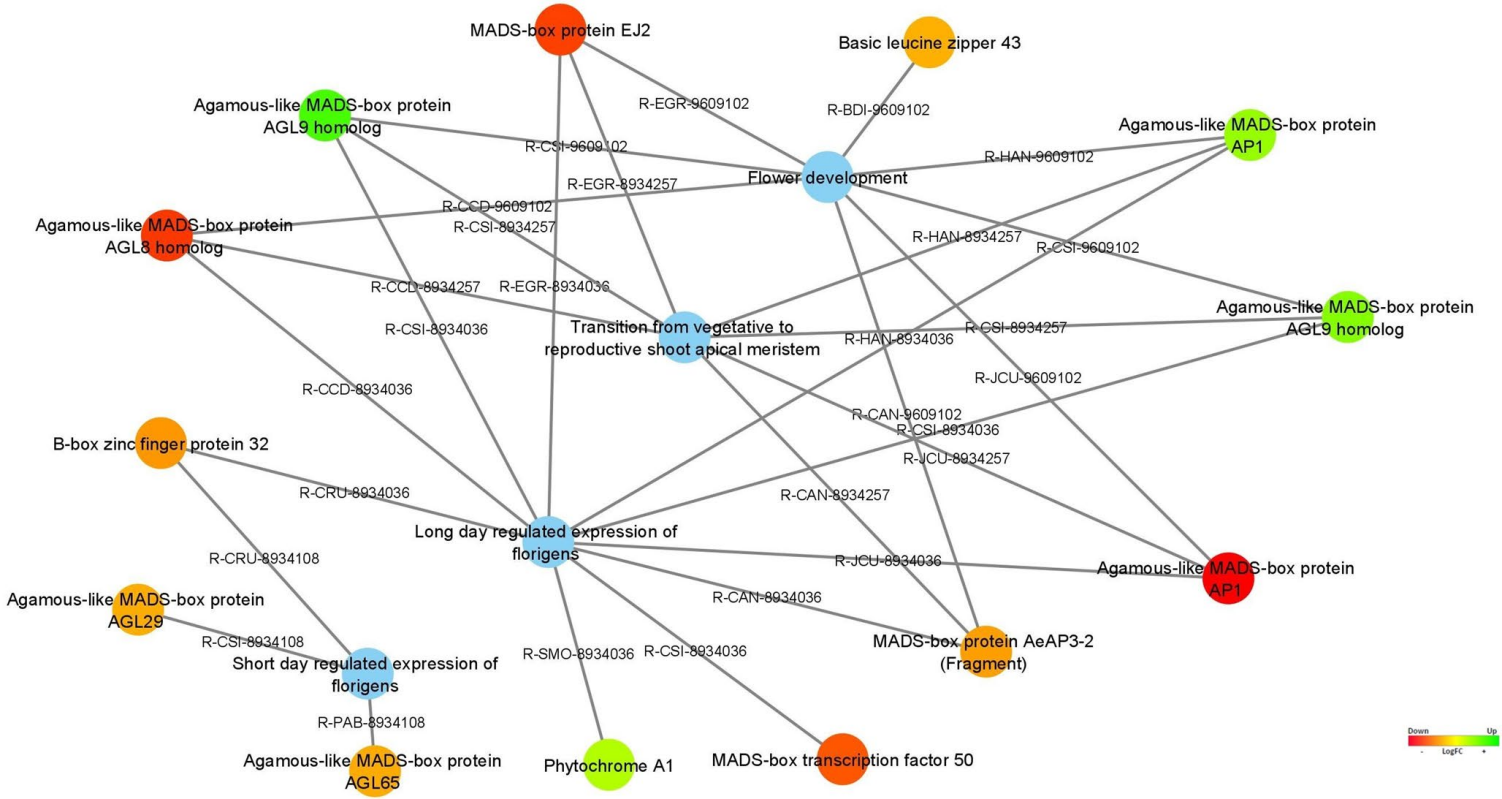
Differentially expressed genes associated with flower development and the transition from vegetative to reproductive shoot apical meristem pathways are represented as the blue node. The expression values were colored based on the RGB scale that goes from the most down-regulated (more expressed in stage 4) in red, to the most up-regulated (more expressed in stage 5/6) in green

Differentially expressed genes associated with the pathways of flower development and the transition from vegetative to reproductive shoot apical meristems are represented in Fig. 6. Within the DEGs identified in *C. cardunculus*, MADS-box protein EJ2 (*EJ2*), Agamous-like MADS-box AP1 (*AP1*), Agamous-like MADS-box AGL8 homolog (*TDR4*), MADS-box protein AeAP3-3 (Fragment) (*AP3-2*), Agamous-like MADS-box protein AGL65 (*AGL65*), Agamous-like MADS-box protein AGL29 (*AGL29*), MADS-box transcription factor 50 (*MADS50*), Basic leucine zipper 43 (*BZIP43*), and B-box zinc finger protein 32 (*BBX32*) were identified as up-regulated in stage 4 (vegetative phase).

**Fig. 7:**
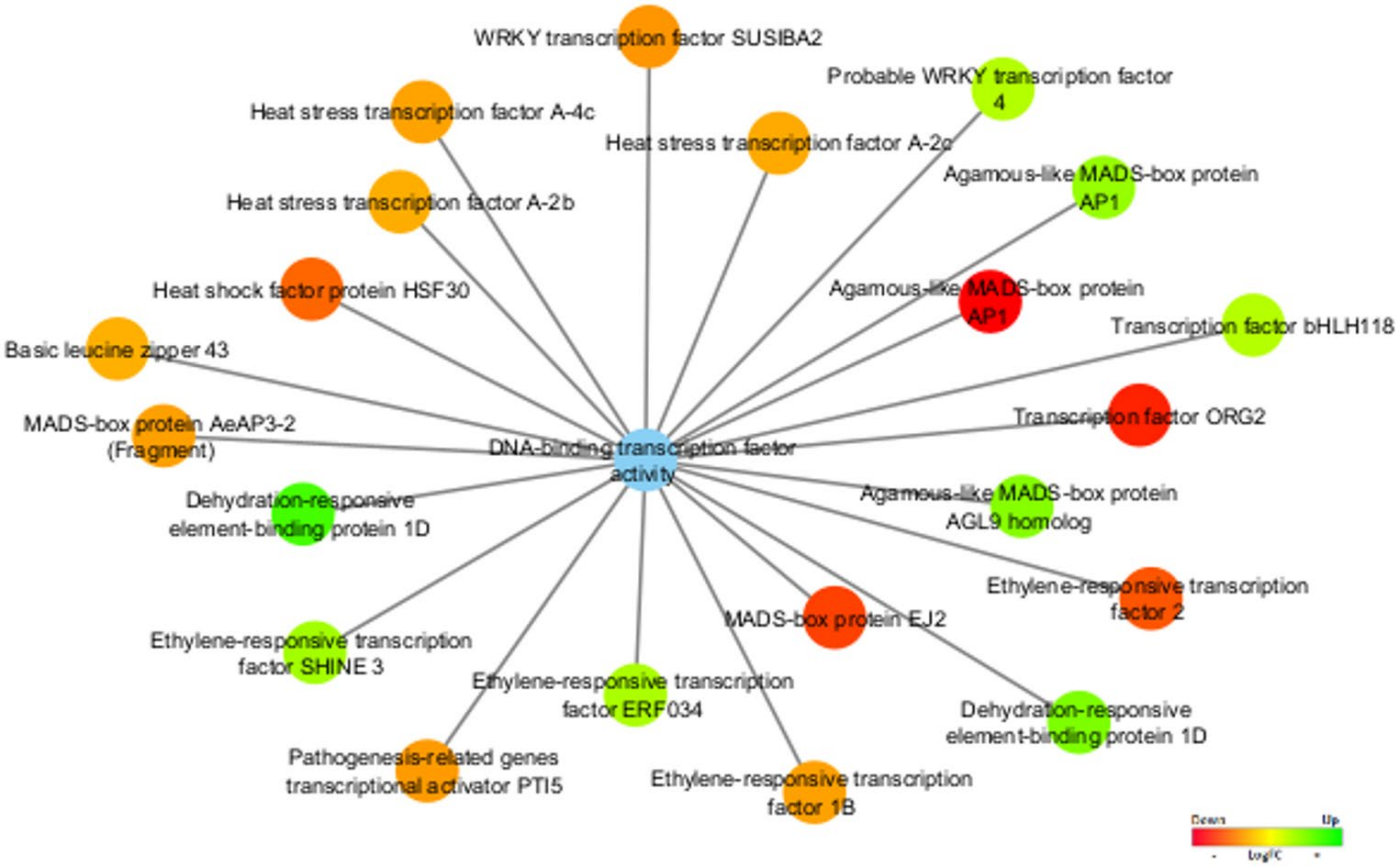
Functional category network of differentially expressed transcription factors annotated with the molecular function DNA-binding transcription factor activity (GO:0003700), represented as the blue node. The expression values were colored based on the RGB scale that goes from the most down-regulated (more expressed in stage 4) in red, to the most up-regulated (more expressed in stage 5/6) in green

Regarding the molecular function DNA binding transcription factor activity (GO:0003700) (Fig. 7) several TFs such as transcription factor ORG2 (*ORG2*), Heat stress transcription factor A-4c (*HSFA4C*), Heat stress transcription factor A-2c (*HSFA2C*), Heat stress transcription factor A-2b (*HSFA2B*), and Ethylene-responsive transcription factor 2 (*ERF2*), Ethylene-responsive transcription factor 1B (*ERF1B*), and WRKY transcription factor SUSIBA2 (*WRKY46*) were found upregulated in stage 4.

**Fig. 8:**
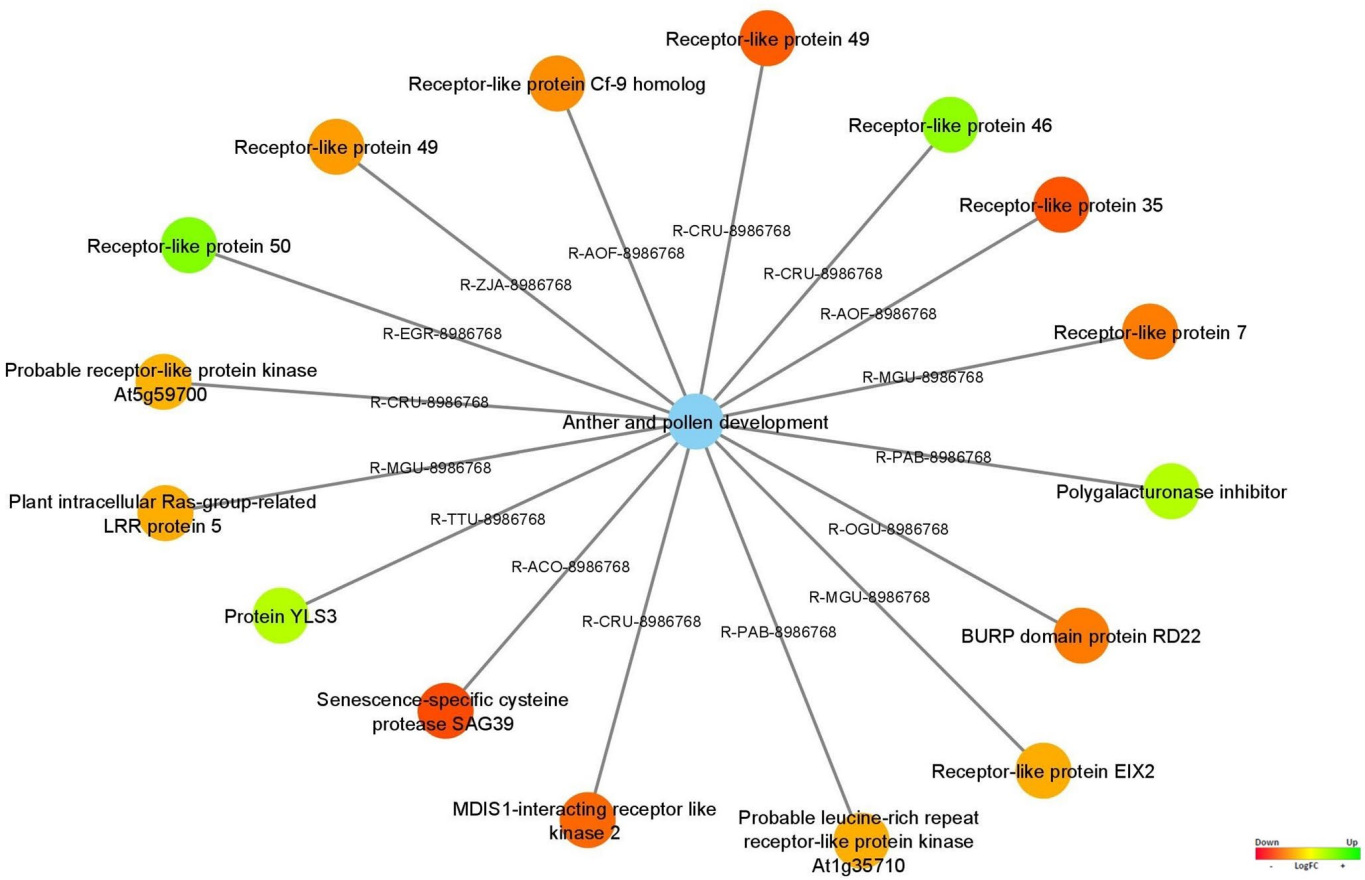
Differentially expressed genes associated with anther and pollen development pathways are represented as the blue node. The expression values were colored based on the RGB scale that goes from the most down-regulated (more expressed in stage 4) in red, to the most up-regulated (more expressed in stage 5/6) in green

Our data revealed also several DEGs up-regulate in stage 4 (vegetative phase) linked to the anther and pollen development pathway (Fig. 8), such as Receptor-like protein 35 (*RLP35*), Receptor-like protein 49 (*RLP49*; codified by two DEGs), Senescence-specific cysteine protease SAG39 (*OsI_14861*), MDIS1-interacting receptor-like kinase 2 (*MIK2*), BURP domain protein RD22 (*RD22*), Receptor-like protein 7 (*RLP7*), Receptor-like protein Cf-9 homolog (*HCR9-0*), Receptor-like protein EIX2 (*EIX2*), Plant intracellular Ras-group-related LRR protein 5 (*IRL5*), Probable leucine-rich repeat receptor-like protein kinase At1g35710 (*At1g35710*), and Probable receptor-like protein kinase At5g59700 (*At5g59700*).

**Fig. 9:**
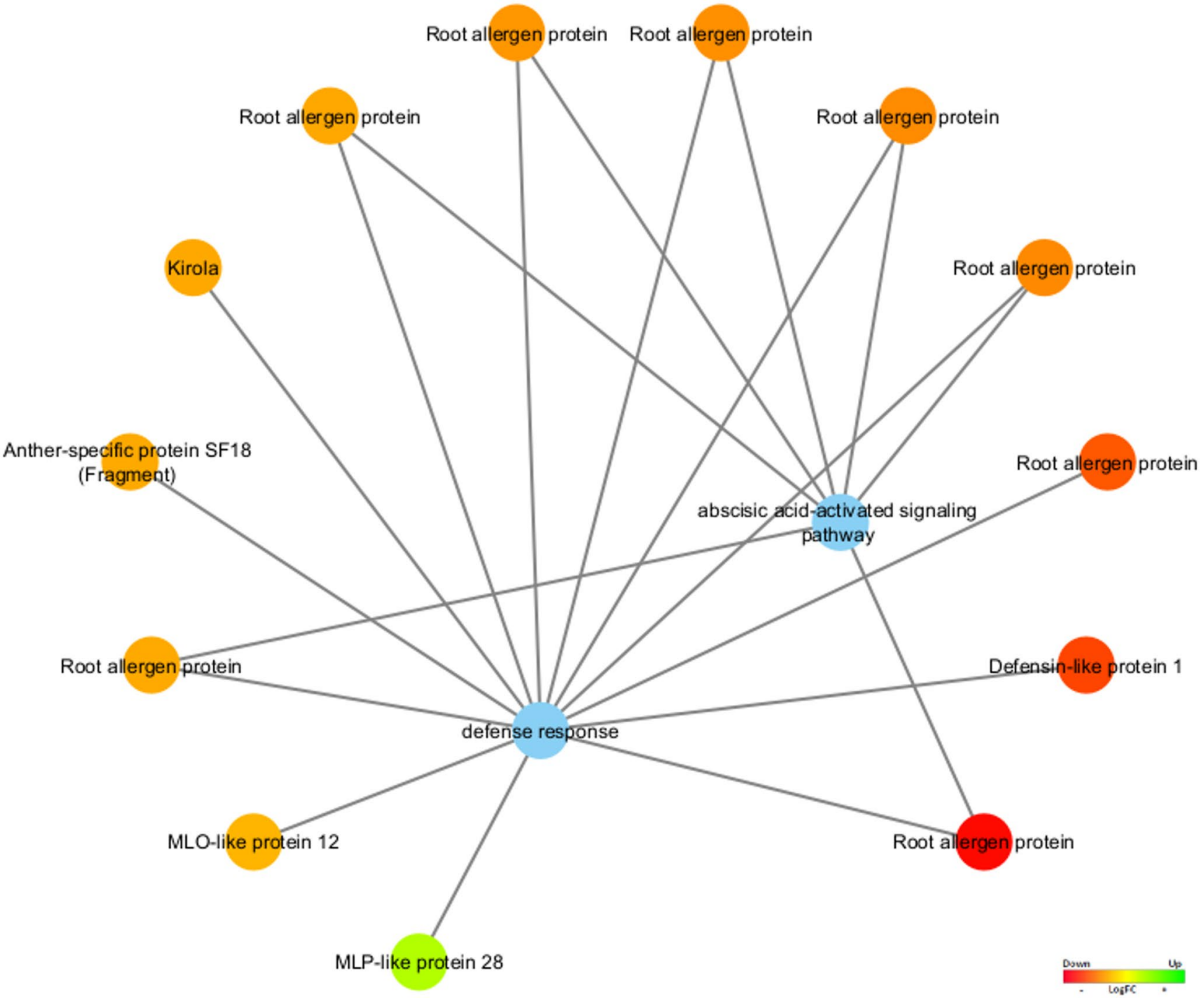
Functional network of differentially expressed genes associated with the biological processes defense response (GO:0006952) and abscisic acid-activated signaling pathway (GO:0009738) are represented as the blue node. The expression values were colored based on the RGB scale that goes from the most down-regulated (more expressed in stage 4) in red, to the most up-regulated (more expressed in stage 5/6) in green

Considering the biological processes, Defense Response (GO:0006952) and absisic acid-activated signaling pathway (GO:0009738), the Defensin-like protein 1, Anther-specific protein SF18 (Fragment), MLO-like protein 12 (*MLO12*), Kirola, and Root allergen protein (codified by eight DEGs) were found upregulated in stage 4 (vegetative stage) (Fig. 9).

**Fig. 10:**
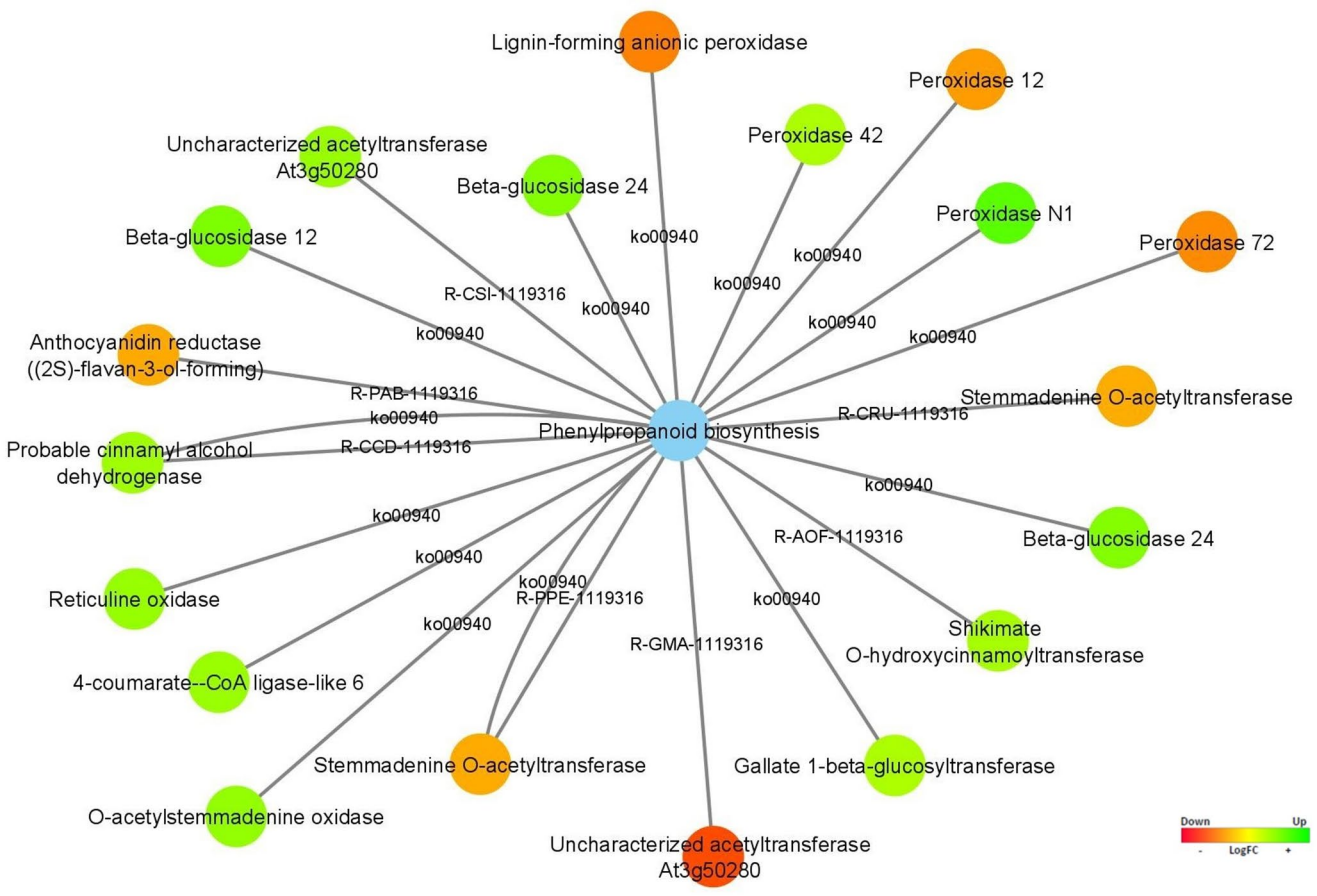
Differentially expressed genes associated with the phenylpropanoid biosynthesis pathway are represented as the blue node. The expression values were colored based on the RGB scale that goes from the most down-regulated (more expressed in stage 4) in red, to the most up-regulated (more expressed in stage 5/6) in green

The phenylpropanoid biosynthesis pathway with the higher number of DEGs associates is represented in Fig. 10, in which the Lignin-forming anionic peroxidase, Peroxidase 72 (*PER72*), Peroxidase 12 (*PER12*), Anthocyanidin reductase ((2S)-flavan-3-ol-forming) (*ANR*), Uncharacterized acetyltransferase At3g50280 (*At3g50280*) and two genes Stemmadenine O-acetyltransferase (AT) were identified as upregulated in stage 4 (vegetative stage), playing various roles in plant biology, including lignin formation, flavonoid synthesis, and acetyltransferase activity.

**Fig. 11:**
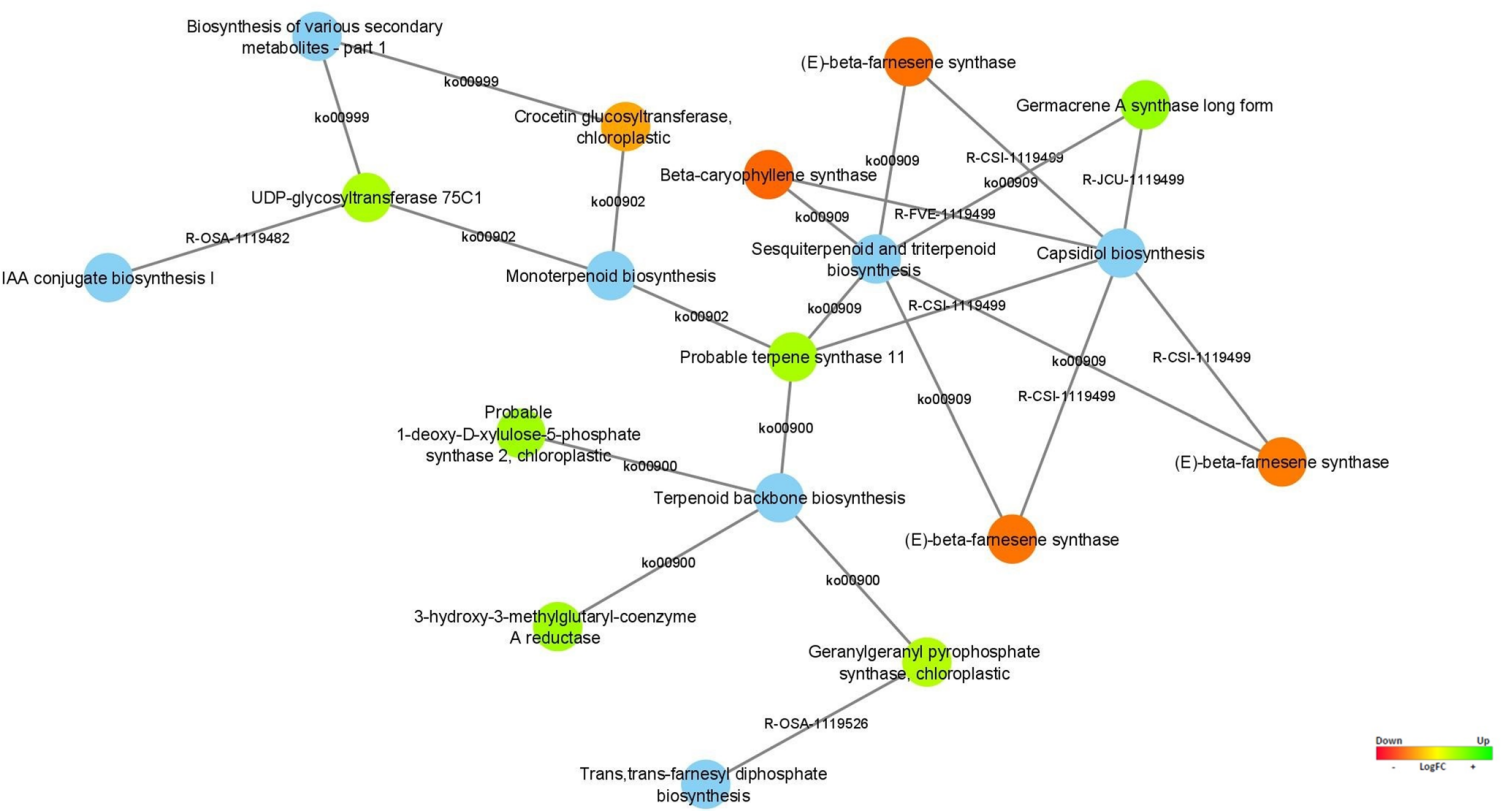
Differentially expressed genes associated with the sesquiterpene lactones biosynthetic pathway are represented as the blue node. The expression values were colored based on the RGB scale that goes from the most down-regulated (more expressed in stage 4) in red, to the most up-regulated (more expressed in stage 5/6) in green

As regards the sesquiterpene lactones biosynthetic pathway (Fig. 11), the Crocetin glucosyltransferase, chloroplastic (*UGT75L6*), Beta-caryophyllene synthase, and (E)-beta-farnesene synthase (codified by three DEGs) were upregulated in stage 4 (vegetative stage).

### Genes highly up-regulated in the reproductive stage (stage 5/6)

Within the pathways of the flower developmental and transition from vegetative to reproductive shoot apical meristem (Fig. 6), several genes were upregulated in stages 5/6, such as Agamous-like MADS-box protein AP1 (*AP1*), Agamous-like MADS-box protein AGL9 homolog (*AGL9*; *FBP2*; codified by two DEGs), and Phytochrome A1 (*PHYA1*).

Regarding the molecular function, DNA binding transcription factor activity (GO:0003700), several TF upregulated in stages 5/6 were identified (Fig. 7), such as Ethylene-responsive transcription factor ERF034 (*ERF034*), Ethylene-responsive transcription factor SHINE 3 (*HN3*), Probable WRKY transcription factor 4, Transcription factor bHLH118, and Dehydration-responsive element-binding protein 1D (*DREB1D*; codified by two DEGs).

The DEGs associated with anther and pollen development pathway, Receptor-like protein 50 (*RLP50*), Receptor-like protein 46 (*RLP46*), Polygalacturonase inhibitor (*pgip*), and Protein YLS3 (*YLS3*) were identified as upregulated in stage 5/6 (reproductive stage) (Fig. 8).

MLP-like protein 28 (*MLP28*), related to the defense response (GO:0006952) (Fig. 9), was identified as up-regulated in stage 5/6 (reproductive stage) in *C. cardunculus*.

From the phenylpropanoid biosynthesis pathway (Fig. 10), Peroxidase N1 (*poxN1*), Beta-glucosidase 12 (*BGLU12*), two genes Beta-glucosidase 24 (*BGLU24*), O-acetylstemmadenine oxidase (*ASO*), Uncharacterized acetyltransferase At3g50280, 4-coumarate-CoA ligase-like 6 (*4CLL6*), Probable cinnamyl alcohol dehydrogenase (*CAD2*), Shikimate O-hydroxycinnamoyltransferase (*HST*), Reticuline oxidase (*BBE1*), Peroxidase 42 (*PER42*), and Gallate 1-beta-glucosyltransferase (*UGT84A13*) were up-regulated in stages 5/6 (reproductive stage).

As regards the sesquiterpene lactones biosynthetic pathway (Fig. 11), the UDP-glycosyltransferase 75C1 (*UGT75C1*), Probable terpene synthase 11 (*TPS11*), Probable 1-deoxy-D-xylulose-5-phosphate synthase 2 chloroplastic (*Os07g0190000*), 3-hydroxy-3-methylglutaryl-coenzyme A reductase, Geranylgeranyl pyrophosphate synthase chloroplastic (*GGPS1*), Germacrene A synthase long form were upregulated in stage 5/6 (reproductive stage).

### Phylogenetic relationships of the analyzed proteins

**Fig. 12:**
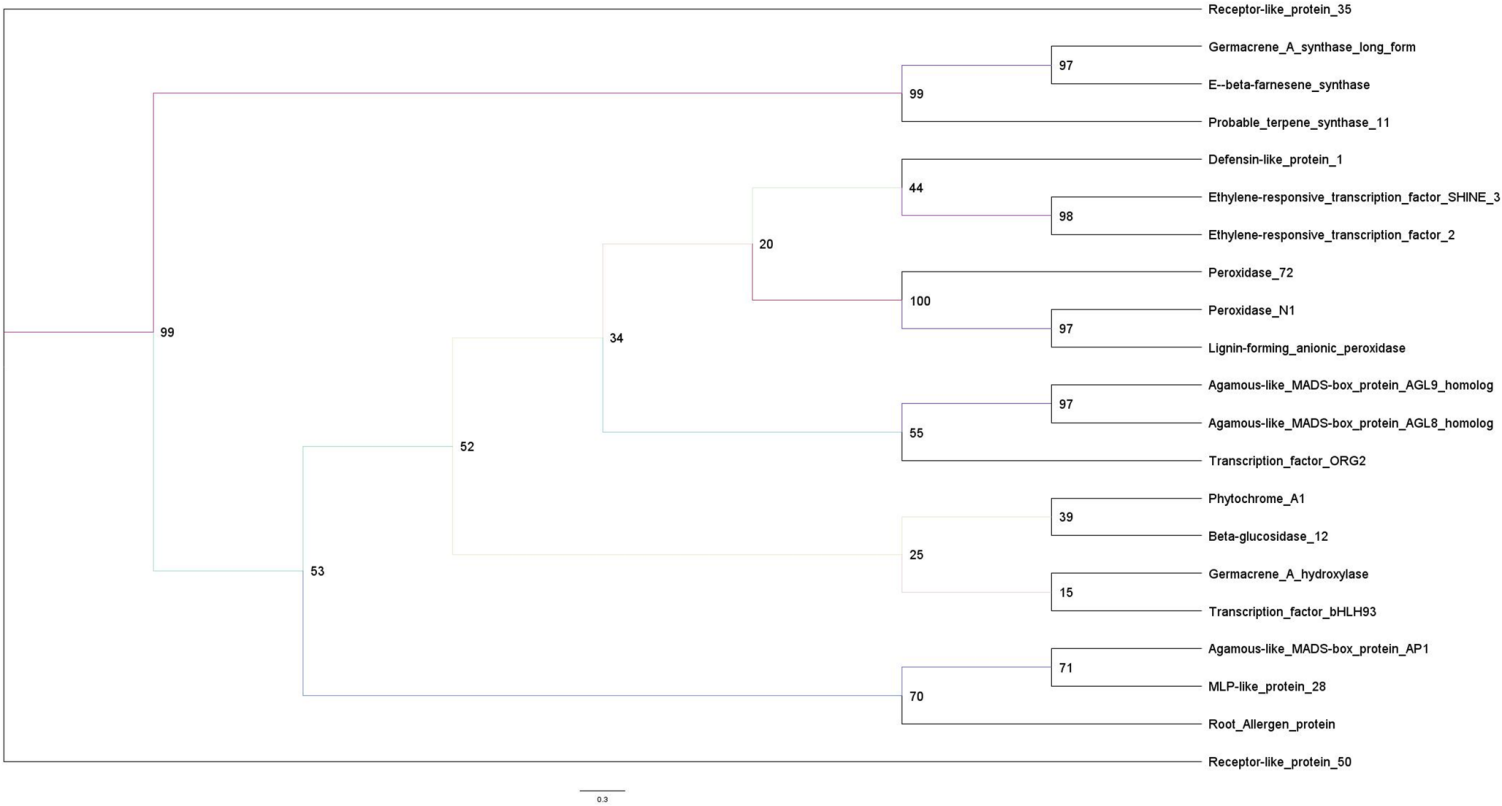
Maximum likelihood phylogenetic tree of the 21 biologically relevant DEGs associated with the vegetative-to-reproductive transition constructed from the MAFFT alignment using IQ-TREE. Bootstrap support values (1000 replicates) are indicated at the nodes. The scale bar represents the number of amino acid substitutions per site

The phylogenetic analysis of the 21 biological relevant DEGs (Table S4) associated with the vegetative-to-reproductive transition revealed a clear clustering according to functional families (Fig. 12). Terpene synthases (*Germacrene A synthase*, *E-β-farnesene synthase* and *probable terpene synthase 11*) formed a highly supported monophyletic clade with bootstrap values ranging from 97 to 99. The peroxidase proteins (*Peroxidase 72*, *Peroxidase N1* and *lignin-forming anionic peroxidase*) clustered together with very strong support (bootstrap = 97–100), indicating a conserved evolutionary origin.

The MADS-box transcription factors *AGL8* and *AGL9* grouped in a robust clade (bootstrap = 97), while the ethylene-responsive transcription factors *SHINE3* and *ERF2* also clustered with strong support (bootstrap = 98). Deeper nodes connecting the major functional groups showed lower bootstrap values, indicating limited resolution among distantly related protein families across the full set of 21 sequences.

### Identification of conserved protein motifs among transcription factor families, along with promoter motif and cis-regulatory element characterization.

MEME analysis identified three conserved protein motifs among the 21 protein sequences analyzed (Fig. S4). The most significant motif (MEME-1) was 49 amino acids long and displayed a very low E-value (5.1 × 10⁻9), indicating strong statistical support. This motif was detected in two sequences corresponding to MADS-box transcription factor genes (AGL8 and AGL9). The motif is highly enriched in basic residues, particularly lysine (K) and arginine (R), which are characteristic of DNA-binding domains. The second most significant motif (MEME-2), with a length of 24 amino acids and an E-value of 6.9 × 10^−8^, detected in the three peroxidase sequences, indicating their potential involvement in catalytic activity. In addition, the third motif (MEME-3; E-value = 7.9 × 10^−5^) with 46 amino acids was also detected in the two peroxidases (poxN1 and PER72), enriched in alanine (A) and valine (V) residues. The remaining motifs showed weak statistical support and are likely to reflect background sequence variability rather than biologically meaningful features.

De novo motif analysis of the promoter regions of the 21 genes identified three conserved DNA motifs, each comprising 15 nucleotides. These motifs were characterised by a high GC/C content and strongly conserved nucleotide positions in motifs 1 and 2 (E values of 1.6 × 10⁻^4^ and 4.1 × 10⁻^4^, respectively), as well as TA patterns in motif 3 (E value of 6.3 × 10⁻^5^). (Fig. S5).

PlantCARE analysis revealed a diverse set of cis-acting regulatory elements distributed across all seven promoters. Core promoter elements, including TATA-box and CAAT-box, were identified in all sequences, confirming the transcriptional competence of the analyzed regions. In addition, multiple hormone-responsive elements were detected, such as ABRE (abscisic acid responsiveness), AuxRE/TGA-elements (auxin responsiveness), GARE-motif and P-box (gibberellin responsiveness), and ERE (ethylene responsiveness). Several promoters also contained light-responsive elements, including G-box, Box 4, and GT1-motifs, as well as stress-related elements such as ARE, MBS, and TC-rich repeats. Elements associated with developmental regulation, including motifs linked to meristem activity and floral development, were particularly evident in promoters of MADS-box transcription factors.

### Correlation between cynaropicrin accumulation and gene expression

**Fig. 13:**
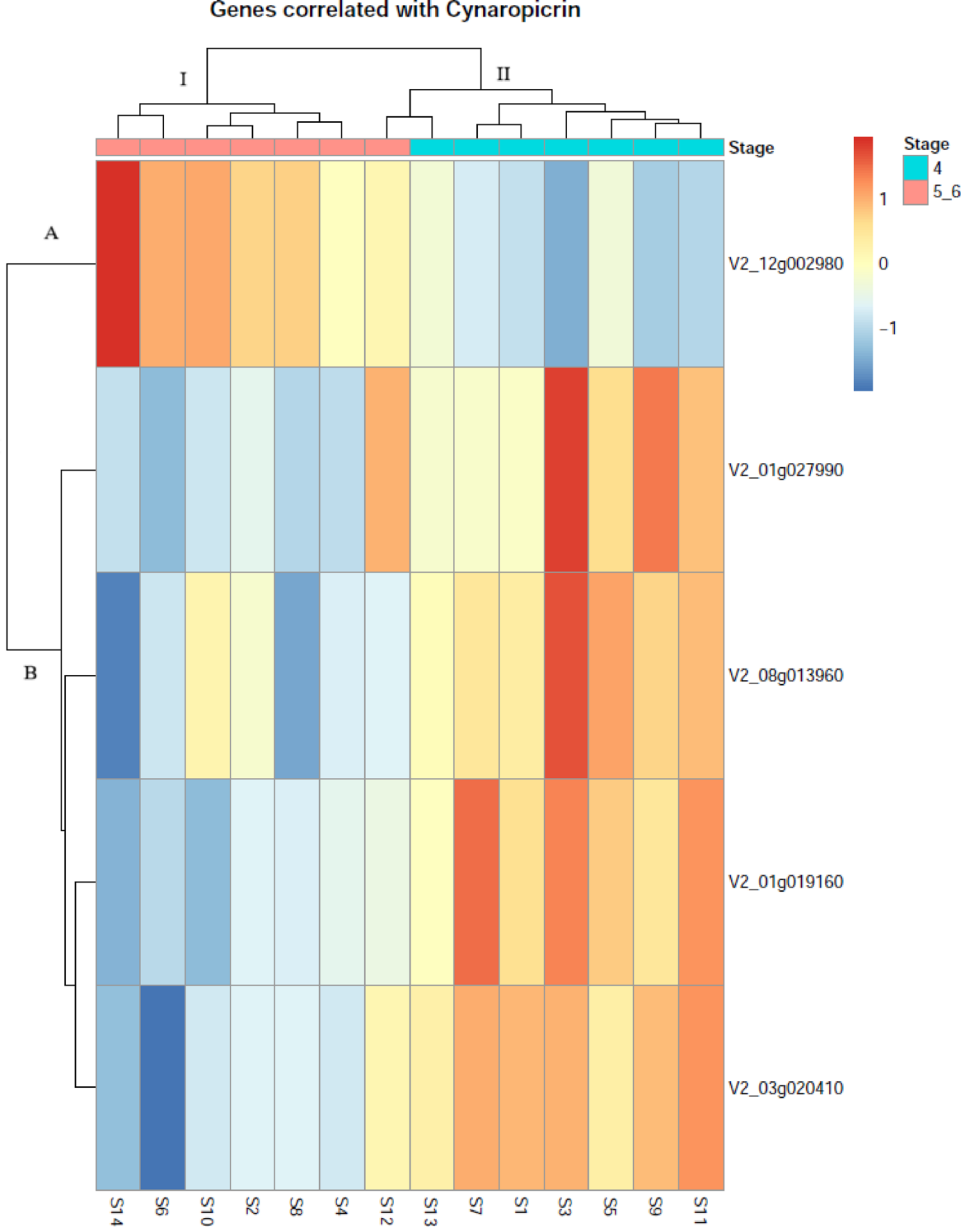
Gene expression patterns associated with cynaropicrin accumulation in *Cynara cardunculus*. Heatmap showing the expression profiles of the five genes significantly correlated with cynaropicrin levels (Spearman’s ρ, FDR < 0.05, |ρ|≥ 0.70) across vegetative (stage 4) and reproductive (stages 5–6) plants. Samples (S1–S14) are ordered according to hierarchical clustering based on gene expression and segregate primarily according to cynaropicrin levels. Expression values are Z-score normalized per gene and represented by a blue to red color scale indicating low to high expression, respectively. The top annotation bar indicates the developmental stage of each sample (vegetative stage 4 and reproductive stages 5–6). Gene hierarchical clustering defines two transcriptional groups: Cluster A, comprising V2_12g002980, which is preferentially expressed in reproductive plants and positively associated with cynaropicrin accumulation; and Cluster B, comprising V2_01g027990, V2_08g013960, V2_01g019160, and V2_03g020410, which show higher expression in vegetative plants and are negatively associated with cynaropicrin levels

To determine whether cynaropicrin accumulation is accompanied by transcriptional changes during development, we performed a Spearman correlation between metabolite levels and the expression of 28,632 transcripts across individual plants representing stages 4 and 5/6. Five distinct transcripts showed significant correlations with cynaropicrin levels (|ρ|≥ 0.70; FDR < 0.05), with correlation coefficients ranging from –0.91 to + 0.87. Thus, the correlation analysis between cynaropicrin accumulation and gene expression across plants with different metabolite levels and developmental stages identified the following five significantly correlated transcripts: *V2_12g002980* (protein of unknown function), *V2_01g027990* (NLP5), *V2_08g013960* (Transcription repressor OFP8), *V2_01g019160* (Thioredoxin-like 1–1, chloroplastic), and *V2_03g020410* (protein of unknown function) (Fig. 13).

Hierarchical clustering (Cluster I and II) separated the samples primarily according to cynaropicrin content, with reproductive plants (stage 5/6) grouping together and displaying higher cynaropicrin levels (Cluster I), while vegetative plants (stage 4) formed a distinct cluster characterized by lower cynaropicrin accumulation (Cluster II). Gene expression analysis revealed two contrasting transcriptional patterns associated with cynaropicrin levels.

The *V2_12g002980* (protein of unknown function) was preferentially expressed in reproductive plants and showed a positive correlation with cynaropicrin accumulation. In contrast, *V2_01g027990* (NLP5), *V2_08g013960* (Transcription repressor OFP8), *V2_01g019160* (Thioredoxin-like 1–1, chloroplastic)*,* and *V2_03g020410* (protein of unknown function) displayed higher expression in vegetative plants and were negatively associated with cynaropicrin levels.

Gene clustering revealed two distinct transcriptional patterns: (i) a gene positively associated with cynaropicrin accumulation and induced during reproductive stage (V2_12g002980); and (ii) a group of genes negatively associated with cynaropicrin levels, preferentially expressed during vegetative stage (*V2_01g027990*, *V2_08g013960*, *V2_01g019160,* and *V2_03g020410)*.

## Discussion

Cardoon, a cross-pollinated perennial species, is characterized by edible inflorescences and serves as a source of floral-based milk coagulants used in traditional cheese production. Additionally, cardoon represents a significant source of secondary metabolites, including phenolics, flavonoids, and sesquiterpene lactones, which contribute to its bioactive properties and biotechnological potential. The present study focused on identifying and interacting with the molecular mechanisms that mediate the vegetative-to-reproductive transition by transcriptomic analysis of two different developmental stages, vegetative and reproductive. RNA-Seq has transformed our ability to examine gene expression dynamics, providing a powerful tool for researchers across many disciplines. Continuous improvements in sequencing technologies and bioinformatics tools are further driving the evolution in this field (Stark et al. 2019).

Using a new sequencing platform, DNBSeq, our study highlighted a high number of DEGs between vegetative and reproductive stages, for the first time in wild cardoon. In our study, a differential gene expression study identified 552 differentially expressed genes (DEGs) (FC > 2), with 321 showing higher expression in the vegetative phase and 231 in the reproductive phase. Of these DEGs, 88% were functionally annotated, identifying several DEGs with functions associated with phenylpropanoid biosynthesis, anther/pollen development, flower development, transcription factors (TFs), defence response, and plant growth regulators have been identified. For the growth and development of plants, the temporal regulation of gene expression is essential, and its understanding contributes to revealing the molecular mechanisms underpinning every developmental phase.

### Morphological and chemical evaluation

In March, *C. cardunculus* genotypes demonstrated a state of vegetative growth phase 4, characterized by adult leaves' full expansion and maturation, with no signs of inflorescence emergence. According to Archontoulis et al. (2010), the biomass yield during this stage can be very variable depending on the climate conditions.

In May, it was possible to observe the emergence of inflorescences in some of the genotypes sampled. The co-authors also suggest that the inflorescence number in this species may depend on environmental conditions, management, and genetic factors to be triggered (Lee et al. 2023). The plants maintained a constant growth rate during the assessed period. It is also important to note that these analyses were specific to the study's conditions and period, and other variables, such as management and climatic factors, may influence the development of *C. cardunculus* plants.

Cynaropicrin, a sesquiterpene lactone with demonstrated anti-inflammatory, antimicrobial, and anticancer properties (Eljounaidi et al. 2015; Ramos et al. 2017), has been also associated with some physiological functions in plants, including defense against insects and pathogens (Elsebai et al. 2016). The contrasting profiles of cynaropicrin observed from vegetative to reproductive stages in our study suggest a potential involvement of this sesquiterpene lactone in the flowering and capitulum formation in cardoon, offering some insights into the biochemical changes associated with flowering and capitulum formation in *C. cardunculus*.

### Novel transcription factor in the regulation of flower development

The transition from vegetative to reproductive shoot apical meristem marks the shift from producing leaves and stems to producing flowers (Blázquez et al. 2001), a process regulated by genetic mechanisms triggered by internal and external signals (Chávez-Hernández et al. 2022). In *C. cardunculus*, floral development is influenced by environmental, genetic, and management factors, resulting in variations in flowering time and the number of inflorescences (Archontoulis et al. 2010; Lee et al. 2023). Transcriptomic studies have been essential to understanding the molecular mechanisms underlying flower development, highlighting the crucial role of transcription factors (TFs) in regulating gene expression.

TFs are proteins that bind to DNA and regulate the transcription of genes involved in plant development (Joshi et al. 2016). Based on the results of Puglia et al. (2020), two-third of the differentially expressed genes (DEGs) identified during capitulum development were TFs. According to our results, 53 DEGs were annotated to the TF category and classified into 8 TF families, with HSF, MADS-box, and bHLH being the most prominent. The MADS-box family (Fig. 7), one of the largest and most studied, plays a central role in floral meristem identity and the determination of floral organs (Schwarz-Sommer et al. 1990; Wang et al. 2019). Overexpression of these genes in the vegetative stage often results in early flowering (Blázquez et al. 2001).

*EJ2* gene coding for MADS-box protein EJ2 up-regulated in cardoon vegetative state, has been already described as particularly active during the reproductive phase in *Solanum lycopersicum* and plays a central role in various aspects of this process, which range from the control of meristem identity to the development of the floral organs, the development/ripening, and abscission of fruit and plays a role in the branching process of the inflorescence (Soyk et al. 2017). Other MAD-box genes have been described in several studies. MADS-box transcription factor 50 regulates flowering time and can stimulate the floral transition stage as well as internode elongation in *A. thaliana* (Tadege et al. 2003; Lee et al. 2004). The Agamous-like MADS-box protein AGL8 homolog (up-regulated in vegetative stage in cardoon) in *A. thaliana*, plays a crucial role in the regulation of floral organ identity and flowering time (Yanofsky et al. 1990; Ferrándiz et al. 2000), while MADS-box protein AeAP3-2 (up-regulated transcript in vegetative stage in cardoon) is involved in flower development in tomatoes and *A. thaliana* (Jack et al. 1992; De Martino et al. 2006). The Agamous-like MADS-box protein AGL65 (*AGL65*) is involved in the regulation of pollen maturation at the late stages of pollen development and pollen tube growth (Adamczyk and Fernandez 2009), while the Agamous-like MADS-box protein AGL29 (*AGL29*) is expressed in pollen and during embryogenesis, expressed in the chalazal endosperm (Kofuji et al. 2003; Bemer et al. 2010).

Additionally, genes codifying for Agamous-like MADS-box protein AP1 (codified by two DEGs, one up and one down-regulated in the reproductive stage), Agamous-like MADS-box protein AGL9 homolog (codified by two DEGs), and Phytochrome A1 were upregulated in the reproductive stage. The Agamous-like MADS-box protein AP1 in *R. chinensis* was also reported as an important gene in the regulation of the development of floral organs, such as petals and stamens, by activating or repressing the expression of downstream genes (Han et al. 2019). The *AGL9* is active in inflorescence development and floral organogenesis (Bonhomme et al. 1997) and Phytochrome A1 is a regulatory photoreceptor that exists in two forms that are reversibly interconvertible by light.

In addition to *MADS-box*, other TF families, such as *ERF*, *bZIP*, *WRKY*, and *bHLH*, are also associated with floral development (Fig. 7). Members of these families were previously described as linked to reproductive development in other plant species, such as *A. thaliana*, *O. sativa*, and *N. tabacum* (Nakano et al. 2006; Li et al. 2018; Hao et al. 2021). The *AP2/ERF* family (33% in our data), for example, is involved in stress responses, development, and hormonal signaling (Feng et al. 2020; Wu et al. 2022). Transcription factors such as *ERF2*, *ERF034*, and *SHINE 3* regulate physiological processes, including fruit ripening, senescence, and responses to biotic and abiotic stresses (Fujimoto et al. 2000; Aharoni et al. 2004; Wu et al. 2022). One of the biggest transcription factor gene families in *A. thaliana* and *C. Cardunculus* var. *altilis* is the basic *helix-loop-helix* (*bHLH*) transcription factor family (Puglia et al. 2020; Hao et al. 2021). The *bHLH* family, in turn, is linked to light signaling, regulation of flowering time, and stress responses (Sun et al. 2018), with members like *ORG2* playing roles in multiple biological processes (Heim et al. 2003; Kang et al. 2003; Wang et al. 2007).

Another TF family associated with the molecular function of DNA-binding transcription factor activity is *WRKY*, which is involved in many physiological processes in plants, including growth, development, and responses to both biotic and abiotic stress (Guo et al. 2019). The Probable *WRKY* transcription factor 4 plays a role in plant defense and stress responses. In *A. thaliana*, it is expressed in young, mature, and senescent leaves, and it is induced by biotic and abiotic stresses (Robatzek and Somssich 2001; Yu et al. 2001; Lai et al. 2008; Yamasaki et al. 2012). These TFs and *MADS-box* coordinate a complex signaling network that regulates floral development and plant adaptation to varying environmental conditions.

### Regulation patterns of other DEGs directly linked to anther and pollen development

Anther and pollen development are essential processes embedded in the reproductive phase of flowering plants (angiosperms) and involve a series of complex molecular and cellular events, including cell differentiation, division, and maturation (Pearce et al. 2015).

Receptor-like kinases (RLKs) are key proteins in plants, mediating signal transduction and responses to environmental cues like hormones, pathogens, and stress (Mizuno et al. 2007). Notable *RLK* genes differently expressed in our study include homologs to *At1g35710* and *At5g59700*, and *MIK2* (encodes the MDIS1-interacting receptor-like kinase 2), which regulates growth, development, and stress responses, and is involved in pollen tube perception.

Also, Receptor-like proteins (RLPs), which are vital for plant defense and development, act as molecular sensors to external signals. In this study, several *RLPs*, such as *RLP35*, *RLP49*, *RLP50*, and *RLP46*, were identified as differentially expressed during anther and pollen development (Fig. 8). The observed differential expression of these *RLPs* aligns with their hypothesized roles in cell signaling during anther maturation, pollen development, and possibly self-incompatibility in *C. cardunculus* (Scaglione et al. 2016).

*SAG39* (codified for Senescence-specific cysteine protease SAG39) was up-regulated in stage 4, while *YLS3* and *pgip* (codified for Protein YLS3 and Polygalacturonase inhibitor, respectively) were up-regulated in stages 5/6. Senescence-specific cysteine protease SAG39 plays a role in developmental senescence, apoptosis, and stress responses (Liu et al. 2010). Protein YLS3 is expressed in expanding leaves and sepals, triggered by abscisic acid (Yoshida et al. 2001; Kobayashi et al. 2011), while *polygalacturonase inhibitor* (*pgip*) enhances plant resistance to fungal attacks by inhibiting cell wall penetration (Howell and Davis 2005). These results demonstrate the complex molecular processes that underlie stress reactions and plant reproduction.

### Defense response

In response to herbivory, plants use a variety of morphological, biochemical, and molecular defense mechanisms to mitigate the impacts of the attack. Both direct and indirect defensive mechanisms facilitate the complex and ever-changing biochemical defense systems against herbivores. The defensive chemicals impact the eating, growth, and survival of herbivores and are either constitutively produced or released in reaction to plant harm. Furthermore, vegetation releases volatile chemical compounds that draw the herbivores' natural predators. These tactics can function separately or in tandem with one another (War et al. 2012).

For example, Defensin-like protein 1, highly represented in stage 4 in our study (Fig. 9), plays an antimicrobial activity by altering fungal membranes (Thevissen et al. 2000). Similarly, root allergen proteins, encoded by eight DEGs in cardoon, can trigger allergic reactions, and their expression varies with environmental and genetic factors (Bonini et al. 1994; Falcon and Caoili 2023). During the reproductive stage, cardoon upregulates *MLP28*, responsible for coding a protein involved in stress responses and disease resistance, which is also linked to the jasmonic acid signaling pathway (Song et al. 2020).

During the reproductive phase, the plant devotes energy to producing flowers and seeds. This energy redistribution can make the plant more sensitive to herbivores, diseases, and other environmental challenges, despite its antibacterial and insecticidal properties (Kant et al. 2015; Quiroz-Pacheco et al. 2020).

### Regulation Patterns of DEGs Directly Linked to Phenylpropanoid Biosynthesis

The phenylpropanoid biosynthesis pathway is a complex metabolic pathway responsible for producing various secondary metabolites in plants, such as phenolic compounds including flavonoids, lignans, and other metabolites (Fraser and Chapple 2011). Our study identified several associated DEGs encoding peroxidases from the phenylpropanoid biosynthesis pathway (Fig. 10), where 60% of these transcripts (*Lignin-forming anionic peroxidase; PER 72; PER 12*) were up-regulated in the plant's vegetative stage (stage 4). One of the many enzymes found in nature that belongs to the class of oxidoreductases is peroxidase. Interaction with hydrogen peroxide and related compounds can accelerate the oxidation of various organic and inorganic substrates. Owing to their broad range of catalytic activity, peroxidases can be used in chemical synthesis, the elimination of phenolic compounds and peroxides, and, considering recent research, the breakdown of mycotoxin (de Oliveira et al. 2021). With potential uses in tobacco, tomato, and sweetgum plants, anionic peroxidases are particularly important in lignin formation and plant development (Lagrimini 1993), with homologs like *PER 72* and *PER 12* in Arabidopsis linked to abiotic and biotic stress responses (Schenk et al. 2000; Fowler and Thomashow 2002). *PER 72* is slightly expressed in roots and induced by cold, while *PER 12* is up-regulated in roots and leaves and expressed in early seed development (Girke et al. 2000). *PER 42*, another peroxidase, may act as a heat shock-like defense protein, with high expression in roots (Schaffer et al. 2001). In tobacco, *peroxidase N1* (*poxN1*) is highly expressed in roots and minimally in lower leaves (Hiraga et al. 2000). However, this study is the first to investigate the presence of these genes in *C. cardunculus.*

*Beta-glucosidase* transcripts (*BGLU12*, *BGLU24* two isoforms) are highly enriched during the reproductive stage in cardoon, playing roles in phytohormone activation, defense, and cell-wall processes (Cairns and Esen 2010). Research in rice highlights *BGLU24's* role in seed germination and its interaction with phytohormones like IAA and ABA, suggesting similar functions in cardoon (Ren et al. 2019). Additionally, studies on *C. cardunculus* var. *altilis* reveal that genes like *Shikimate O-hydroxycinnamoyltransferase* (*HCT*) and *4-coumarate-CoA ligase* (*4CL*) are up-regulated during inflorescence development, with *4CLL6* and *HST* showing increased expression in the reproductive stage (Puglia et al. 2020).

The higher expression of these genes during the reproductive stage suggests their involvement in various processes associated with flowering, fruiting, seed development, and putative plant defense mechanisms at this stage of the plant's life cycle.

### Metabolite production

In plants, defense responses are crucial to their survival and protection against various biotic and abiotic stresses (Gull et al. 2019). These defense mechanisms are often triggered by the recognition of pathogens, such as bacteria, fungi, viruses, and herbivores (War et al. 2012). The responses involve complex signaling pathways and produce various metabolites that contribute to the plant's defense arsenal. Understanding the growth profile of *C. cardunculus* leaves is crucial for crop production to increase sesquiterpene lactone yield.

Terpenoids, a diverse class of secondary metabolites, play a vital role in plant defense and other biological functions (Singh and Sharma 2015). Their biosynthesis begins with the formation of a terpenoid backbone via the mevalonate or methylerythritol phosphate (MEP) pathways. Key intermediates like trans,trans-farnesyl diphosphate (FPP) serve as precursors for sesquiterpenoids and triterpenoids, which are synthesized by enzymes such as sesquiterpene synthases and oxidosqualene cyclases (LI et al. 2023a, b). The Probable Terpene Synthase 11 (*TPS11*) is a protein-coding gene that is involved in the biosynthesis of terpenes (Li et al. 2022). *TPS11*, identified in our study as upregulated in the reproductive stage (Fig. 11), has been identified in various plant species, including *A. thaliana* (Aubourg et al. 2002; Tholl and Lee 2011), *Medicago truncatula* (Parker et al. 2014), and *tomato* (Falara et al. 2011). It is primarily expressed in the glandular trichomes of these plants, which are specialized structures that produce and secrete secondary metabolites such as terpenes (Parker et al. 2014). Overall, the TPS11 protein plays an important role in the biosynthesis of sesquiterpenes in plants, which have a variety of functions, including defense against herbivores and pathogens, as well as attraction of pollinators and seed dispersers.

UDP-glycosyltransferase 75C1 acts on both abscisic acid (ABA) auxin (IAA) biosynthesis and was found highly expressed during fruit ripening and flower development in tomatoes (Sun et al. 2017), supporting the up-regulation of UDP-glycosyltransferase 75C1 in stage 5/6 of cardoon development.

Another important enzyme, beta-caryophyllene synthase which is highly represented in stage 4 of wild cardoon, has previously been identified in *C. cardunculus* L. var. *scolymus* heads (Laghezza Masci et al. 2024) is responsible for producing the sesquiterpene beta-caryophyllene, which is known by its anti-inflammatory and anti-carcinogenic properties. Its expression varies across plant tissues and decreases with seedling age (Gu et al. 2002; Olofsson et al. 2011). Similarly, (E)-beta-farnesene synthase converts FPP into (E)-beta-farnesene, a volatile compound that acts as a signaling molecule during stress or herbivory and influences insect behavior (Bhatia et al. 2015). In wild cardoon, three *(E)-beta-farnesene synthase* genes were found upregulated during the vegetative stage.

Other DEGs responsible for codifying the protein Germacrene A synthase long form were present in our results, which is involved in sesquiterpene lactone biosynthesis, and produces exclusively ( +)-germacrene A (GAS). The GAS previously isolated from globe artichoke participates in the first stage of cynaropicrin biosynthesis, where the expression analysis revealed a link between GAS expression level and cynaropicrin concentration (Menin et al. 2012). On the other hand, it was mainly found expressed in roots, head core tissue, and green and etiolated seedlings of chicory, while its expression was lower in green leaves (Bouwmeester et al. 2002). This finding supports our results, in which GAS is upregulated in the reproductive stage, a period with a higher production of cynaropicrin in wild cardoon leaves.

Our results show that the outcomes derived from the various plants during the two months of collection exhibit significant variation in cynaropicrin levels, concerning both genotype and collection timeframe. This difference suggests that the production of cynaropicrin in plants is activated during the vegetative to reproductive transition in *C. cardunculus*, which can be influenced by environmental, physiological, and genetic factors (Paulino et al. 2023). The elevated presence of cynaropicrin can act as a deterrent against herbivores, functioning as a natural insecticide. According to our genetic results, this gene is upregulated in the reproductive stage, a period with a higher production of cynaropicrin in *C. cardunculus* leaves. Later, germacrene A oxidase (GAO) (upregulated in the vegetative stage in our results; Table S3) and costunolide synthase (COS) genes, also involved in cynaropicrin biosynthesis, were identified and functionally described in *C. cardunculus* (Eljounaidi et al. 2014).

Overall, the regulation of terpenoid biosynthesis in *C. cardunculus* is tightly linked to the vegetative-to-reproductive transition, highlighting the importance of terpenoids in plant defense and their potential applications in agriculture and medicine.

### Phylogenetic relationships and conserved protein motifs of differentially expressed genes with cis-regulatory element profiling

Phylogenetic analysis of the 21 DEGs revealed a clear clustering according to major functional families, indicating strong evolutionary conservation and shared ancestry. In particular, terpene synthases, peroxidases, and MADS-box transcription factors formed well-supported clades, reflecting their conserved biological roles across plant lineages. The deep evolutionary conservation and diversification of MADS-box genes across land plants has been extensively documented and underscores their fundamental role in developmental regulation and transcriptional control (Parenicová et al. 2003; Qiu et al. 2023; Zhang et al. 2024).

The close clustering observed among terpene synthases suggests coordinated evolutionary trajectories linked to terpenoid biosynthesis, a key component of plant secondary metabolism and stress adaptation. Similarly, the peroxidase clade exhibited strong phylogenetic support, highlighting the conservation of enzymes involved in oxidative stress responses, cell wall modification, and lignification. Together, these phylogenetic patterns indicate that the selected genes are embedded within conserved functional families, supporting their biological relevance in growth, development, and environmental responses.

The integration of protein motif analysis with phylogenetic relationships revealed a strong correspondence between evolutionary clustering and motif composition. Protein motif analysis identified three conserved motifs across the 21 proteins, mapped specifically to phylogenetically clusters, indicating preservation of key functional domains likely linked to protein–protein interactions and catalytic activity. The most significant motif, a long and highly conserved region enriched in charged residues, corresponds to the canonical MADS DNA-binding domain, supporting their common evolutionary origin and conserved regulatory function of TF family (Theissen et al. 2000; Smaczniak et al. 2012). This reinforces the importance of the transcriptional regulation during the vegetative-to-reproductive transition. Likewise, motifs enriched in peroxidase proteins were restricted to peroxidase sequences, consistent with conserved residues linked to enzymatic activity.

De novo motif discovery identified conserved sequence patterns shared among promoters, while PlantCARE annotation detected cis-regulatory elements associated with hormonal signaling, light responsiveness, and stress responses. The coexistence of conserved de novo motifs and annotated cis-elements supports coordinated regulation of transcription factor genes by developmental and environmental cues, a regulatory strategy widely reported in plants (Lescot et al. 2002; Bailey et al. 2009; Sheshadri et al. 2016). Notably, the enrichment of hormone and stress responsive cis-elements, suggests that these genes may function as regulatory nodes integrating environmental signaling with secondary metabolic pathways (Toledo-Ortiz et al. 2003; Nakano et al. 2006; Licausi et al. 2013).

### Transcriptional control of cynaropicrin biosynthesis during plant development

The integration of gene expression with cynaropicrin accumulation across developmental stages shows that cynaropicrin biosynthesis in *C. cardunculus* is predominantly associated with the reproductive phase (stages 5/6). This pattern is consistent with the developmental regulation of sesquiterpene lactones reported in Asteraceae reported by Liu et al. 2011. In *Cynara cardunculus*, a coordinated increase in sesquiterpene lactones during later developmental stages has also been described, supporting this developmental control in the species (Eljounaidi et al. 2015).

Among the genes correlated with cynaropicrin accumulation, *V2_12g002980* (protein of unknown function) display preferential expression in reproductive plants and showed a positive association with cynaropicrin levels, suggesting a direct involvement in the biosynthetic pathway or in regulatory steps linked to enhanced metabolite production. Such transcriptional induction of pathway related genes during reproductive development has been widely reported for sesquiterpene lactone biosynthesis (Nguyen et al. 2010).

In contrast, *V2_01g027990* (*Nodule inception-like proteins, NLP5*), *V2_08g013960* (*OVATE gene, OFP8*), *V2_01g019160* (*Thioredoxin-like 1–1, chloroplastic*, *TLL1*)*,* and *V2_03g020410* (protein of unknown function) were more highly expressed during vegetative stage (stage 4) and exhibited negative correlations with cynaropicrin accumulation (Gaudinier et al. 2018; Hernández-Reyes et al. 2022; Yang et al. 2016). The transcription factor *NLP5*, described as being involved in nitrogen signaling (Gaudinier et al. 2018; Hernández-Reyes et al. 2022), suggests a role in coordinating nitrogen status with metabolic allocation during cardoon vegetative growth. Meanwhile, the transcription repressor *OFP8* (Yang et al. 2016), known to act as a transcriptional repressor, may limit the activation of secondary metabolic pathways during early developmental stages. Moreover, the role of the *thioredoxin-like 1–1, chloroplastic*, *TTL1* in the adaptation of the *Arabidopsis thaliana* plant to osmotic stress and in the control of root growth has been described (Lakhssassi et al 2012).

The expression pattern identified in our study also suggests that these genes may be involved in alternative branches of terpenoid metabolism or in the allocation of metabolic precursors toward other pathways during vegetative growth, rather than directly contributing to cynaropicrin biosynthesis.

## Conclusion

Cardoon, a cross-pollinated perennial crop, is a major source of secondary metabolites such as phenylpropanoids, flavonoids, and sesquiterpenes lactones. Its production and regulation can be boosted during inflorescence emergence/development.

The main goal of the current study was to determine the molecular mechanisms involved in the vegetative and reproductive phases using a comparative transcriptome analysis. This work uncovered candidate transcripts not previously associated with flower development or secondary metabolites.

Based on the results presented, significant differences were observed in the morphological growth, cynaropicrin levels, and gene expression of *C. cardunculus* across its different phenological stages. During the vegetative stage (BBCH 4), the plants exhibited considerable leaf area and variable height, but no inflorescences, highlighting a phase of intense vegetative growth. In contrast, at the reproductive stage (BBCH 5/6), inflorescence development was observed, with greater dispersion between plants, suggesting a lack of complete synchronization in flowering. Cynaropicrin quantification showed a significant increase in this compound’s content from March to May, which could reflect changes in environmental conditions and plant development. This compound, important for both pharmaceutical and food industries, was found to be more concentrated in May, indicating that the reproductive stage might be crucial for its accumulation. Transcriptome analysis revealed a substantial number of differentially expressed genes (DEGs), with 321 genes being more expressed in the vegetative stage and 231 in the reproductive stage. DEGs related to secondary metabolite biosynthesis, such as phenylpropanoid pathways, were particularly prominent in both stages, with metabolic pathways related to pollen development and defense responses more active in the vegetative stage. In contrast, genes linked to terpene biosynthesis and flower development were more expressed in the reproductive stage. Novel transcription factors in the regulation of flower development were also identified, with HSF, MADS-box, and bHLH being the most prominent families.

The integrative analysis of the most representative DEGs, revealed coherent regulatory and metabolic modules associated with the floral transition. The coordinated clustering of MADS-box regulators, transcription factors, receptor-like proteins, phenylpropanoid enzymes and sesquiterpene synthases, along with their shared regulatory motifs, indicates that the transition to the reproductive stage is governed by interconnected pathways integrating developmental cues, hormone signaling, and secondary metabolism. This provides a solid molecular framework for understanding the control of flowering and the associated metabolic shifts in Asteraceae.

Overall, this study demonstrates that cynaropicrin accumulation during the transition from stage 4 to stages 5/6 is strongly associated with coordinated transcriptional reprogramming involving both positive and negative regulatory components. The integrative approach adopted here identified a concise and robust set of candidate genes potentially involved in cynaropicrin biosynthesis and regulation, constituting a valuable foundation for future functional and biochemical studies. These findings significantly advance our understanding of sesquiterpene lactone specialization in *C. cardunculus* and open new perspectives for its biotechnological and industrial exploitation.

## Supplementary Information

Below is the link to the electronic supplementary material.

Supplementary Material 1

Supplementary Material 2

Supplementary Material 3

Supplementary Material 4

Figure S1. Cynaropicrin standard curve. Linear relationship between cynaropicrin concentration ([cyn], mg/mL) and instrumental response, described by the regression equation with a coefficient of determination (R² = 0.9965).

Figure S2. Representative HPLC chromatogram of cynaropicrin.

Figure S3. FastQ Screen results after pre-processing. Most reads align uniquely to the *Cynara cardunculus* genome (light blue), with minimal mapping to other reference genomes.

Figure S4. Conserved protein motifs identified by MEME analysis. Conserved amino acid motifs were identified in the analyzed protein set using MEME (v5.5.0) under the ZOOPS model. Sequence logos represent the relative frequency and conservation of amino acids at each position, with letter height proportional to information content (bits). Among the ten motifs detected, Motifs 1–3 showed strong statistical support (E-value < 1e−4) and were considered biologically relevant. Motif 1 (49 aa; E-value = 5.1 × 10^−9^) and Motif 2 (24 aa; E-value = 6.9 × 10^−8^) displayed the highest conservation, while Motif 3 (46 aa; E-value = 7.9 × 10^−5^) showed moderate conservation. Remaining motifs exhibited higher E-values and are likely to represent low-confidence or non-functional patterns.

Figure S5. Identification of conserved motifs in the analyzed promoter regions using the MEME program. Enriched motifs detected in the promoter sequences are shown as sequence logos, in which the height of each letter represents the degree of nucleotide conservation at each position. The analysis was performed using the ZOOPS (zero or one occurrence per sequence) model, considering both DNA strands.

## Data Availability

The data used in this study are part of the BioProject accession number PRJNA975359, available through the NCBI database. The specific BioSample IDs corresponding to each sample are listed in Table 2.
