# Supplementary material for: Unravelling the molecular mechanisms of vegetative-to-reproductive transition in Cynara cardunculus by RNA-Seq analysis
Source: Plant Mol Biol. 2026 Jan 31;116(1):15. doi: 10.1007/s11103-025-01679-2 (PMC12860834; doi:10.1007/s11103-025-01679-2)
Supplement: Supplementary file 1 — Supplementary Material 1 [file 11103_2025_1679_MOESM1_ESM.docx]

Table S1. Summary of morphological evaluation from Cynara cardunculus samples, identified according to ID. Offshoots: number of offshoots in each plant (R1, R2, and R3 represent the different offshoots). Height (cm): height of each offshoot. Foliar area (cm2): overrated area = length x width of Cynara cardunculus leaf. Inflorescence: number of inflorescences in each C. cardunculus offshoot. Nd: not determined.

| **Sample ID** | **Month** | **offshoots** | **Height (cm)** | | | | ***C. cardunculus* Leaf** | | | | | | | | | | **Inflorescence** | | |
| --- | --- | --- | --- | --- | --- | --- | --- | --- | --- | --- | --- | --- | --- | --- | --- | --- | --- | --- | --- |
|  |  |  | **R1** | **R2** | **R3** | **Mean** | **R1** | | | **R2** | | | **R3** | | | **Total Foliar area Mean (cm^2^)** | **R1** | **R2** | **Mean** |
|  |  |  |  |  |  |  | **length (cm)** | **width (cm)** | **foliararea (cm^2^)** | **length (cm)** | **width (cm)** | **foliararea (cm^2^)** | **length (cm)** | **width (cm)** | **foliararea (cm^2^)** |  |  |  |  |
| 1 | March | 2 | 42 | 43 | nd | 43 | 75 | 38 | 2850 | 67 | 35 | 2345 | nd | nd | nd | 1732 | nd | nd | nd |
| 3 | March | 2 | 39 | 21 | nd | 30 | 63 | 39 | 2457 | 54 | 32 | 1728 | nd | nd | nd | 2093 | nd | nd | nd |
| 5 | March | 3 | 51 | 48 | 37 | 45 | 67 | 34 | 2278 | 51 | 31 | 1581 | 75 | 36 | 2700 | 2186 | nd | nd | nd |
| 7 | March | 3 | 32 | 21 | 13 | 22 | 57 | 26 | 1482 | 64 | 33 | 2112 | 54 | 27 | 1458 | 1684 | nd | nd | nd |
| 9 | March | 2 | 47 | 42 | nd | 45 | 68 | 39 | 2652 | 66 | 35 | 2310 | nd | nd | nd | 2481 | nd | nd | nd |
| 11 | March | 1 | 43 | nd | nd | 43 | 56 | 30 | 1680 | nd | nd | nd | nd | nd | nd | 1680 | nd | nd | nd |
| 13 | March | 3 | 106 | 97 | 102 | 102 | 96 | 55 | 5280 | 113 | 49 | 5537 | 84 | 52 | 4368 | 5062 | nd | nd | nd |
| 2 | May | 2 | 83 | 73 | nd | 78 | 64 | 29 | 1856 | 68 | 27 | 1836 | nd | nd | nd | 1231 | 3 | 3 | 6 |
| 4 | May | 2 | 36 | 29 | nd | 33 | 56 | 35 | 1960 | 45 | 23 | 1035 | nd | nd | nd | 1498 | nd | nd | nd |
| 6 | May | 3 | 89 | 58 | 48 | 65 | 98 | 41 | 4018 | 83 | 31 | 2573 | 91 | 35 | 3185 | 3259 | 12 | nd | 12 |
| 8 | May | 3 | 35 | 24 | 21 | 27 | 47 | 30 | 1410 | 52 | 23 | 1196 | 42 | 22 | 924 | 1177 | nd | nd | nd |
| 10 | May | 2 | 64 | 33 | nd | 49 | 61 | 33 | 2013 | 54 | 27 | 1458 | nd | nd | nd | 1736 | 4 | 1 | 5 |
| 12 | May | 1 | 38 | nd | nd | 38 | 47 | 26 | 1222 | nd | nd | nd | nd | nd | nd | 1222 | nd | nd | nd |
| 14 | May | 1 | 142 | nd | nd | 142 | 110 | 56 | 6160 | nd | nd | nd | nd | nd | nd | 6160 | nd | nd | nd |
