# Supplementary material for: Unravelling the molecular mechanisms of vegetative-to-reproductive transition in Cynara cardunculus by RNA-Seq analysis
Source: Plant Mol Biol. 2026 Jan 31;116(1):15. doi: 10.1007/s11103-025-01679-2 (PMC12860834; doi:10.1007/s11103-025-01679-2)
Supplement: Supplementary file 2 — Supplementary Material 2 [file 11103_2025_1679_MOESM2_ESM.docx]

Table S2. The number of genes expressed is identified according to ID and developmental stage. The results were determined using quantification data from htseq-count.

| **Development stage** | **Sample ID** | **Expressed genes (>0)** | **Expressed genes (≥5)** |
| --- | --- | --- | --- |
| Stage 4 | 1 | 21850 | 20316 |
| Stage 4 | 3 | 21728 | 20062 |
| Stage 4 | 5 | 21735 | 20086 |
| Stage 4 | 7 | 21648 | 20036 |
| Stage 4 | 9 | 21836 | 20195 |
| Stage 4 | 11 | 21625 | 19999 |
| Stage 4 | 13 | 21485 | 20152 |
| Stage 5/6 | 2 | 21945 | 20433 |
| Stage 5/6 | 4 | 21719 | 20084 |
| Stage 5/6 | 6 | 21132 | 19523 |
| Stage 5/6 | 8 | 21518 | 20030 |
| Stage 5/6 | 10 | 22177 | 20734 |
| Stage 5/6 | 12 | 20718 | 19172 |
| Stage 5/6 | 14 | 21374 | 19686 |
