# Supplementary material for: Unravelling the molecular mechanisms of vegetative-to-reproductive transition in Cynara cardunculus by RNA-Seq analysis
Source: Plant Mol Biol. 2026 Jan 31;116(1):15. doi: 10.1007/s11103-025-01679-2 (PMC12860834; doi:10.1007/s11103-025-01679-2)
Supplement: Supplementary file 4 — Supplementary Material 4 [file 11103_2025_1679_MOESM4_ESM.docx]

*Table S4. About 21 biological relevant DEGs associated with the vegetative-to-reproductive transition.*

| **Gene ID** | **Gene_description** | **Gene_symbol** |
| --- | --- | --- |
| V2_04g006020 | Agamous-like MADS-box protein AP1 | *AP1* |
| V2_04g006030 | Agamous-like MADS-box protein AGL8 homolog | *TDR4* |
| V2_04g002460 | Agamous-like MADS-box protein AGL9 homolog | *AGL9* |
| V2_02g010990 | Phytochrome A1 | *PHYA1* |
| V2_07g004760 | Transcription factor bHLH93 | *BHLH93* |
| V2_12g007980 | Ethylene-responsive transcription factor 2 | *ERF2* |
| V2_10g000800 | Ethylene-responsive transcription factor SHINE 3 | *HN3* |
| V2_05g006470 | Transcription factor ORG2 | *ORG2* |
| V2_01g003470 | Receptor-like protein 35 | *RLP35* |
| V2_09g003170 | Receptor-like protein 50 | *RLP50* |
| V2_15g015260 | Root allergen protein | *N/A* |
| V2_02g005320 | Defensin-like protein 1 | *N/A* |
| V2_12g002730 | MLP-like protein 28 | *MLP28* |
| V2_08g005920 | Peroxidase N1 | *poxN1* |
| V2_12g004910 | Lignin-forming anionic peroxidase | *N/A* |
| V2_07g005960 | Peroxidase 72 | *PER72* |
| V2_16g010950 | Beta-glucosidase 12 | *BGLU12* |
| V2_05g012370 | Germacrene A hydroxylase | *GAO* |
| V2_01g024140 | Germacrene A synthase long form | *N/A* |
| V2_15g014110 | (E)-beta-farnesene synthase | *CASC125* |
| V2_12g014780 | Probable terpene synthase 11 | *TPS11* |
