# Supplementary figures and images for: Unravelling the molecular mechanisms of vegetative-to-reproductive transition in Cynara cardunculus by RNA-Seq analysis
Source: Plant Mol Biol. 2026 Jan 31;116(1):15. doi: 10.1007/s11103-025-01679-2 (PMC12860834; doi:10.1007/s11103-025-01679-2)

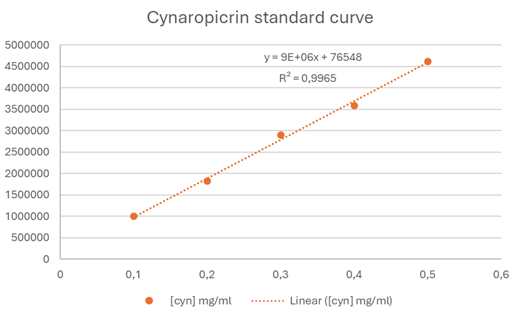

Supplement: Supplementary file 5 — Figure S1. Cynaropicrin standard curve. Linear relationship between cynaropicrin concentration ([cyn], mg/mL) and instrumental response, described by the regression equation with a coefficient of determination (R² = 0.9965). [file 11103_2025_1679_MOESM5_ESM.png]

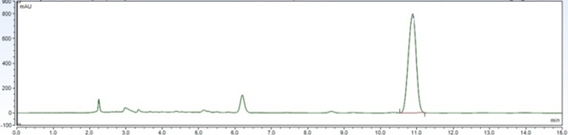

Supplement: Supplementary file 6 — Figure S2. Representative HPLC chromatogram of cynaropicrin. [file 11103_2025_1679_MOESM6_ESM.png]

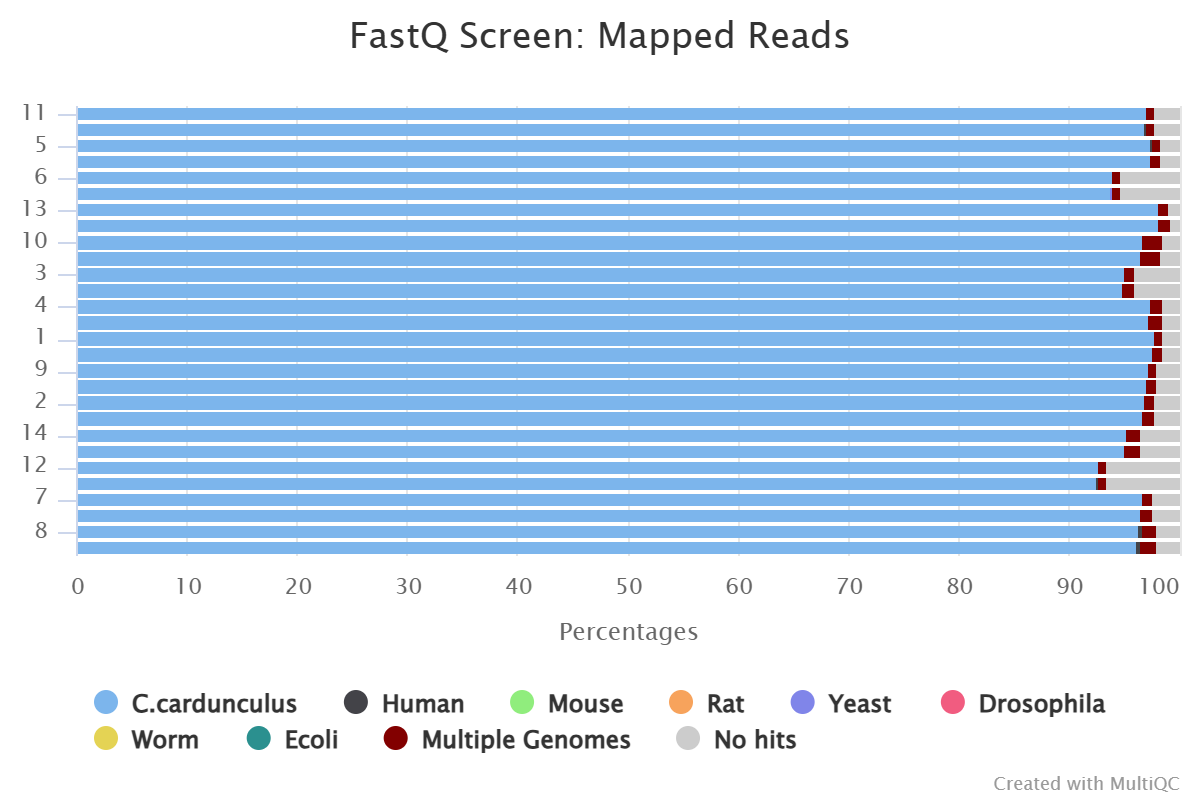

Supplement: Supplementary file 7 — Figure S3. FastQ Screen results after pre-processing. Most reads align uniquely to the Cynara cardunculus genome (light blue), with minimal mapping to other reference genomes. [file 11103_2025_1679_MOESM7_ESM.png]

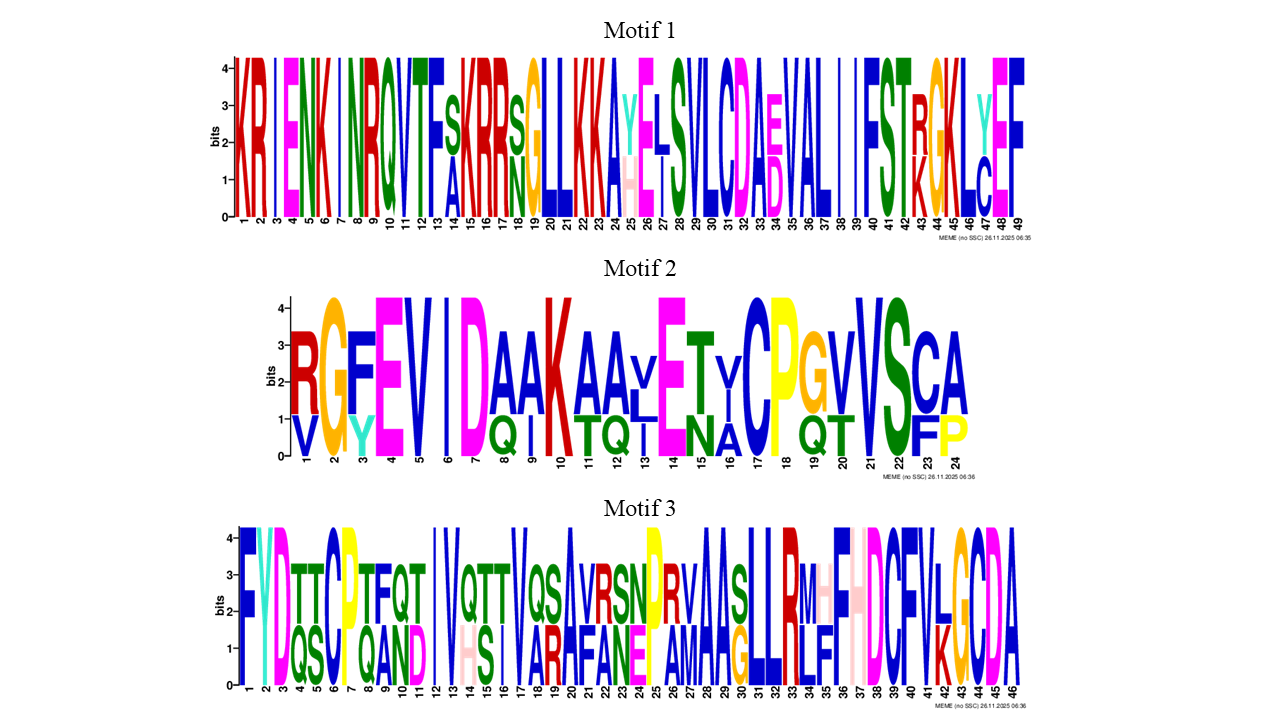

Supplement: Supplementary file 8 — Figure S4. Conserved protein motifs identified by MEME analysis. Conserved amino acid motifs were identified in the analyzed protein set using MEME (v5.5.0) under the ZOOPS model. Sequence logos represent the relative frequency and conservation of amino acids at each position, with letter height proportional to information content (bits). Among the ten motifs detected, Motifs 1–3 showed strong statistical support (E-value < 1e−4) and were considered biologically relevant. Motif 1 (49 aa; E-value = 5.1 × 10^−9^) and Motif 2 (24 aa; E-value = 6.9 × 10^−8^) displayed the highest conservation, while Motif 3 (46 aa; E-value = 7.9 × 10^−5^) showed moderate conservation. Remaining motifs exhibited higher E-values and are likely to represent low-confidence or non-functional patterns. [file 11103_2025_1679_MOESM8_ESM.tif]

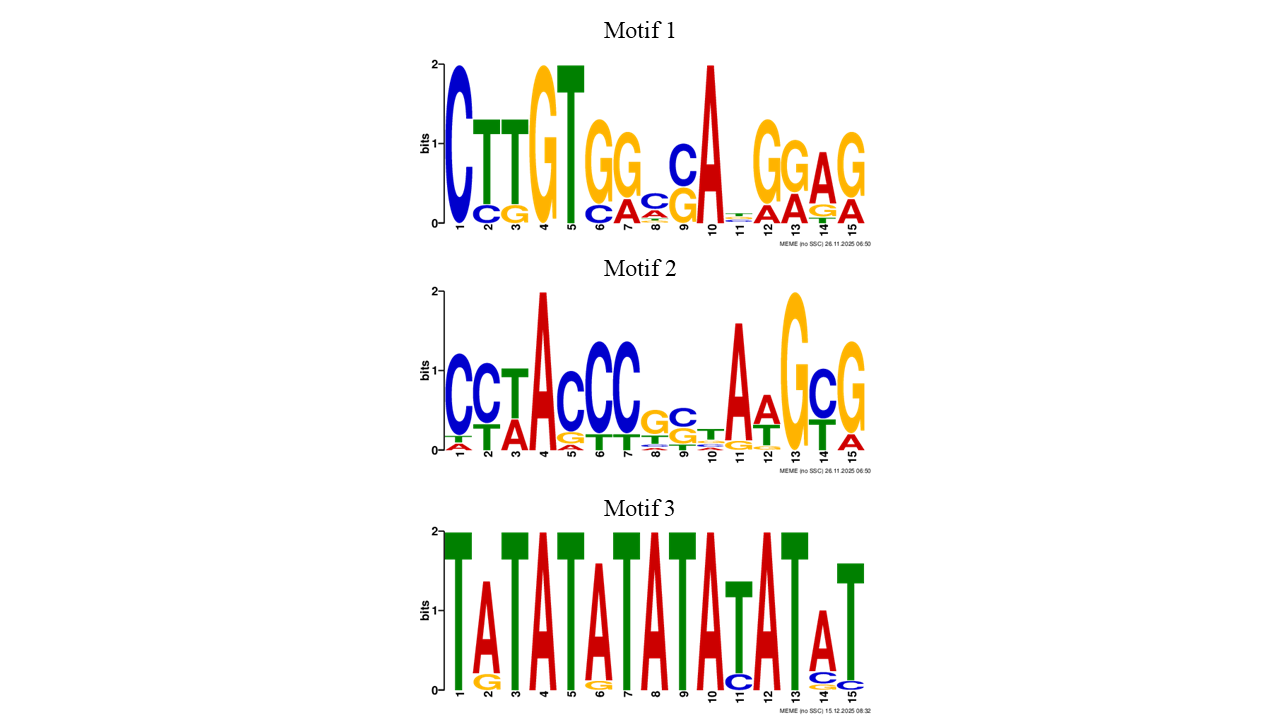

Supplement: Supplementary file 9 — Figure S5. Identification of conserved motifs in the analyzed promoter regions using the MEME program. Enriched motifs detected in the promoter sequences are shown as sequence logos, in which the height of each letter represents the degree of nucleotide conservation at each position. The analysis was performed using the ZOOPS (zero or one occurrence per sequence) model, considering both DNA strands. [file 11103_2025_1679_MOESM9_ESM.tif]
